# Isotopes and Trace Elements as Natal Origin Markers of *Helicoverpa armigera* – An Experimental Model for Biosecurity Pests

**DOI:** 10.1371/journal.pone.0092384

**Published:** 2014-03-24

**Authors:** Peter W. Holder, Karen Armstrong, Robert Van Hale, Marc-Alban Millet, Russell Frew, Timothy J. Clough, Joel A. Baker

**Affiliations:** 1 Bio-Protection Research Centre, Lincoln University, Canterbury, New Zealand; 2 Department of Chemistry, Otago University, Dunedin, New Zealand; 3 School of Geography Environment and Earth Sciences, Victoria University of Wellington, Wellington, New Zealand; 4 Department of Soil and Physical Sciences, Lincoln University, Canterbury, New Zealand; Natural Resources Canada, Canada

## Abstract

Protecting a nation's primary production sector and natural estate is heavily dependent on the ability to determine the risk presented by incursions of exotic insect species. Identifying the geographic origin of such biosecurity breaches can be crucial in determining this risk and directing the appropriate operational responses and eradication campaigns, as well as ascertaining incursion pathways. Reading natural abundance biogeochemical markers using mass spectrometry is a powerful tool for tracing ecological pathways as well as provenance determination of commercial products and items of forensic interest. However, application of these methods to trace insects has been underutilised to date and our understanding in this field is still in a phase of basic development. In addition, biogeochemical markers have never been considered in the atypical situation of a biosecurity incursion, where sample sizes are often small, and of unknown geographic origin and plant host. These constraints effectively confound the interpretation of the one or two isotope geo-location markers systems that are currently used, which are therefore unlikely to achieve the level of provenance resolution required in biosecurity interceptions. Here, a novel approach is taken to evaluate the potential for provenance resolution of insect samples through multiple biogeochemical markers. The international pest, *Helicoverpa armigera*, has been used as a model species to assess the validity of using naturally occurring δ^2^H, ^87^Sr/^86^Sr, ^207^Pb/^206^Pb and ^208^Pb/^206^Pb isotope ratios and trace element concentration signatures from single moth specimens for regional assignment to natal origin. None of the biogeochemical markers selected were individually able to separate moths from the different experimental regions (150–3000 km apart). Conversely, using multivariate analysis, the region of origin was correctly identified for approximately 75% of individual *H. armigera* samples. The geographic resolution demonstrated with this approach has considerable potential for biosecurity as well as other disciplines including forensics, ecology and pest management.

## Introduction

Biosecurity encompasses the provision of services that minimise the impact of exotic pest species on a nation's economy, environment and public health. In agriculturally based economies, such as that of New Zealand, biosecurity systems protect industries worth billions of dollars against constant risk of exotic pest introduction [1], which have large direct and indirect financial costs [2]. As biosecurity risks escalate with the increased international mobility of people and trade products [3], these systems need to become more efficient. This includes an emerging requirement to ascertain the natal geographic origin of intercepted exotic pests, as this is commonly unknown for organisms that are detected in surveillance networks. Such a capability could be used to differentiate between non-established individuals and members of established (locally breeding) populations. This information would direct appropriate response actions in post-border investigations and eradication campaigns, as an unestablished exotic pest requires a much lower scale response than an established population. Similarly, knowing immediate prior origins can help verify a region's pest free status for specific high impact pests, and so maintain trade access [4] by confirming intercepted individuals as vagrant rather than locally established. Point-of-origin data could also be used to identify biosecurity risk pathways and so inform biosecurity policy for pre-border protection.

Although tracing the geographical origins and dispersal of insects are important components within many aspects of entomological science, there are currently no suitable methods available that can determine the immediate origin of biosecurity interceptions. Tracing the dispersal of insects by classical methods, such as mark and recapture, is clearly unavailable for biosecurity investigation, where it is necessary to interpret naturally occurring, unlabelled specimens – as is also the case for many other ecological and pest management studies [5]. Likewise, genetic methods that use the similarity of heritable DNA markers to infer invasion histories or original sources of an introduction are inappropriate for resolving such recent and dynamic relationships [6]. DNA markers can help to assign an individual to a likely population, and therefore by inference the geographic place at which that genetic population is known to occur [7], [8]; however, they cannot discriminate a new invader from a less recent one given the intergenerational time necessary for DNA mutations to be acquired. Consequently the DNA signature of an insect that had just arrived (F_0_, i.e. of exotic origin and non-established) would look the same as one that could putatively have arrived from the same place one or more generations prior (>F1, i.e. of local origin); therefore an intercept could not be distinguished as having just arrived or not.

On the other hand, stable isotope ratio and trace element concentration signatures (‘biogeochemical markers’) can be direct indicators of provenance. These markers are not heritable, but are intrinsically incorporated into the tissues of all members of a population via their food and water sources as the organisms develop [9]. Hence, the markers that vary spatially due to differences in geology [10], elevation and climate [11], such as ^87^Sr/^86^Sr and δ^2^H, may provide the desired understanding of the immediate origin of intercepted samples and distinguish an insect as either F_0_ or ≥F_1_ with respect to establishment status. Various natural abundance biogeochemical markers have been successfully applied to track a wide range of dispersing organisms and items of commercial or forensic interest [12]. However, to date, such markers have been underutilised for provenance determination in entomology, and our understanding in this field is still in a phase of basic development. Early investigations considered concentrations of the small series of common elements able to be analysed with the spectrometry techniques available at the time, (e.g., P, S, Cl, K, Ca, Fe, Cu, Zn) [13]–[16]. However, these elements are biologically active [17] and thus their concentrations are subject to variation linked to physiological differences between individual insects. Consquently, these markers were confounded by polyphagy, adult feeding and gender differences affecting elemental expression, which masked the point-of-origin signals [18], [19]. More recently, stable isotopes have been considered and spatial separation of insect populations across continental δ^2^H and δ^13^C contours has been demonstrated [20], [21]. However, the scale of resolution from these light elements can be too coarse for confident provenance determination [22], [23], with often insufficient difference between study areas and/or the within-region environmentally driven variation in signal being greater than the between-region differences [24]. This is of particular consequence in forensic or biosecurity applications, where the typically small sample sizes impede statistically confident provenance assignment [25]. The specific impetus for the current study was the inability to determine the origin of two important biosecurity pests collected post-border in Auckland, New Zealand in 2005 and 2006 – painted apple moth (*Teia anartoides*, Lymantriidae) and fall web worm (*Hyphantria cunea*, Arctiidae). Based on the successful elucidation of monarch butterfly migration routes [26], provenance assignment for these Auckland incursions using δ^2^H and δ^13^C was attempted [27]. However, interpretation of the results was inconclusive, as the accuracy and limitations of this methodology were unknown in a biosecurity context. In contrast to the Hobson et al. [26] study, that used a single host-plant system within a pre-defined time and space, the Auckland specimens belong to polyphagous species and were accidentally introduced; as is typical with biosecurity interceptions. Therefore, these insects were from an unknown and unpredictable host, place and point in time, which impeded isoscape-to-insect corrections.

A number of reviews have proposed that provenance discrimination may be enhanced by multivariate analyses of several markers [12], [28]–[31], although few studies have empirically tested this, e.g., [32]–[34]. Consequently, the research hypothesis for this study was that the level of spatial resolution and confidence in provenance assignment for biosecurity samples could be improved by combining the continental scale, temperature-linked distribution patterns of δ^2^H, with the finer spatial scale of geological markers such as the isotopes of Sr and Pb and trace element concentrations. In testing this hypothesis, we also assess both the practical feasibility of such a method, and whether the regional spatial resolution achieved is sufficient for biosecurity applications.

## Methods

### Model insect and host plant system

*Helicoverpa armigera* (Hübner 1805) [Lepidoptera: Noctuidae: Heliothinae] (tomato fruit worm) was used as an experimental model of an invasive pest. The fundamental biological parameters of this species are well understood, it is readily field collectable and its pan-global distribution facilitated geographically extensive sample collection in locations appropriate to the research objectives. Further, *H. armigera* is a major pest of food, fibre, oil and ornamental crop plants [35]. There is an ongoing interest in elucidating this species migratory patterns and population dynamics, with view to improving the effectiveness of pest management strategies against it [36], [37]. *Zea mays* (‘corn’) was selected as the most suitable model plant for the inter-regional comparison. It is grown extensively in the areas of research interest and is a productive *H. armigera* host, on which this insect has comparatively low levels of parasitism. Further, *Zea mays* does not support the morphologically similar species *Helicoverpa punctigera* (Wallengren), facilitating the field collection of the correct species.

### Study design and sample collection

**Figure 1:**
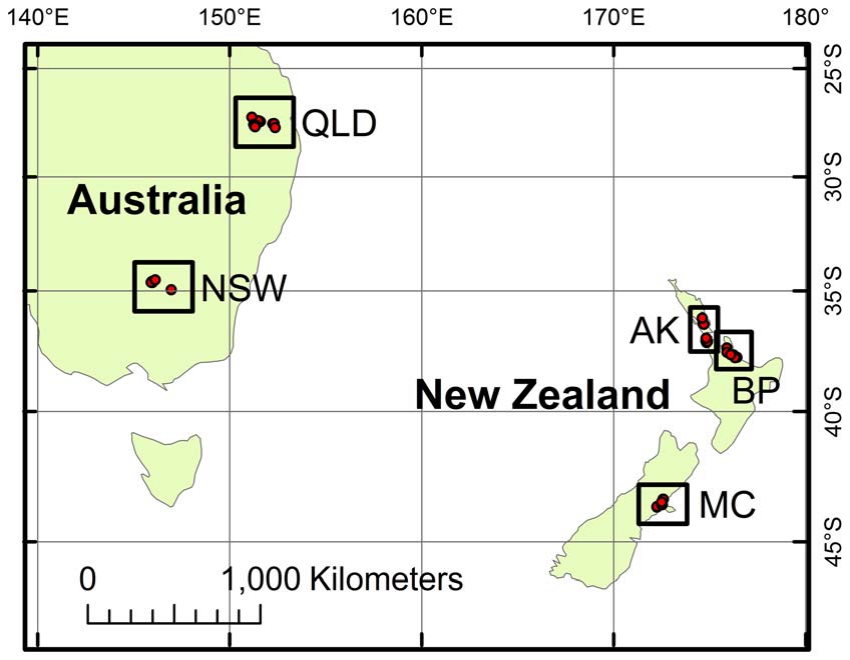
Australasian regions used to test biogeochemical markers for provenance assignment of *H. armigera*. These regions represent biogeochemical contrasts and similarities, and they are important areas with regard to biosecurity for both Australia and New Zealand. MC  =  Mid Canterbury, BP  =  Bay of Plenty, AK  =  Auckland, New Zealand; NSW  =  Wagga Wagga, QLD  =  Toowoomba, Australia.

The bio-geographical regions of Mid-Canterbury (MC), Bay of Plenty (BP), and Auckland (AK) in New Zealand, and the corn growing areas around Toowoomba (Queensland – QLD) and Wagga Wagga (New South Wales – NSW) in Australia were used for comparison (Figure 1). These regions were selected because they represent geological and climatic contrasts and similarities, the model insect-host system occurs in them all, and they are important areas with regard to New Zealand biosecurity [38], [39].

Sample collection was carried out over January – May (southern hemisphere late summer) in two consecutive years, 2008 and 2009, in order to also examine inter-year variation. The collection dates were adjusted between the years so as to occur at the same development phase of both the corn crop (beginning of Kernel Dent Stage) and *H. armigera* phenology (pre-diapause late instar larvae and pupae) in the two summers. *Helicoverpa armigera* were collected from a minimum of 12 separate sites (paddocks) at each of the five different regions, however, some sites did not yield any adult moth samples, and others provided several (Table 1).

**Table 1:** Number of *H. armigera* adult moths reared (*n*) and number of sites represented.

		MC	BP	AK	NSW	QLD
**2008**	*n*	17	8	24	22	26
	sites	9	6	11	7	9
**2009**	*n*	44	38	61	58	36
	sites	8	13	12	11	9

To ensure the specimens were from known locations, and to avoid the potential influence of multiple host plant sources, late instar *H. armigera* larvae were collected from corn cobs for subsequent rearing, and/or pupae were excavated from under the host plants at each site. The larvae were reared on their original cob, until pupation. These and the excavated pupae were held and emerged under a constant 25°C, 16:8 h light: dark regime. Emerged moths were held without food or water for four days, to avoid the influence of adult feeding and to allow the wings to complete sclerotization, then euthanized and stored frozen (−20°C), dry, for later identification and analysis.

### Insect identification

The identification of the collected moths was confirmed as *H. armigera* by screening to genus using fore-wing patterns and to species or species group using hind-wing markings [40]. For specimens where species determination was not possible using exterior morphological examination the identification was confirmed using characteristics of the genitalia [41] and DNA bar-coding [42] (GenBank accession numbers KF661352 – KF661389).

### Ethics statement

No animal care approval was required for the collection and handling of *H. armigera*. The specimens were collected on commercial properties with the permission of the land owners. Live samples from Australia were transferred to New Zealand quarantine facilities under a ‘Permit to Import Live Animals’ from Biosecurity New Zealand (Ministry of Primary Industries) (Permit numbers 2008033670, 2009036197).

### Sample preparation and chemical analyses

Each moth was partitioned to provide samples for the various analyses. A set of wings was dissected for δ^2^H analysis and the remainder of the moth bodies were used for Sr and Pb isotope and trace element concentration analyses.

Samples used for δ^2^H measurement were washed three times with a solution of 2∶1 chloroform: methanol to remove oils and then air dried for 12 h. Six, ≈200 μg pieces (three replicate pairs) were dissected from the distal costal section of the wing and loosely crimped into 3×5 mm silver elemental analyzer cups (OEA Laboratories, UK). Samples were then equilibrated in a pair of static, sealable chambers with one of two water vapours (−258.0‰ or +60.0‰ VSMOW) at 110 °C for 1 hour with vacuum drying at 110°C before and after equilibration, modified from [43]. δ^2^H measurements were conducted using a vacuum purged Costech Zero Blank autosampler on a Thermo TC/EA coupled to a Thermo Delta V IRMS in continuous flow mode, at Otago University, New Zealand. The raw δ^2^H values were corrected to the nine IAEA-CH-7 reference standards (δ^2^H_VSMOW_ −100.3‰) measured at intervals during each batch. Paired results from the equilibrations with the two waters were used to calculate the non-exchangeable hydrogen isotope ratio using equation 3 of Schimmelmann et al. [44]. KHS (−54.1±0.6‰) [45] was used as the quality assurance standard. Average precision of measurement over the three months that the analyses were carried out was ±0.8 ‰.

In preparation for the solution chemistry used for trace element and Sr–Pb isotope analyses, individual moths were ‘washed’ by passing two 30 second 250 kPa+ streams of high purity N_2_ over them in a filtered chamber, as described by Font et al.[46]. All subsequent specimen handling, chemistry and drying was conducted under ultra-clean conditions, within PicoTrace Class 10 laminar flow workstations. Samples were digested using three Seastar 15 M HNO_3_+30% H_2_O_2_ closed digestion – evaporation cycles in Savillex Teflon beakers at 120°C; then cooled and taken up into solution in 1 M HNO_3_. A weighed aliquot, comprising approximately 20% of this solution, was then subject to trace element analysis, using an Agilent 7500cs ICPMS *via* a Cetac ASX-520 autosampler and a 100 μl/min Microflow nebuliser spray chamber (Victoria University of Wellington Geochemistry Laboratory, New Zealand) (Tables S1 & S2, Figure S1). Element concentrations were determined by bracketing each set of five samples with a multi-element calibration standard solution made up from mono-elemental standard solutions (BDH Laboratory Supplies, England). The remaining portion of the solutions were dried down for Sr and Pb separation column procedures, as described by Pin & Bassin [47] and Baker et al. [48] respectively, using 1 ml pipette tips fitted with pre-cleaned 30 μm pore-size polypropylene frits and pre-cleaned Sr Spec (Eichrom Technologies, IL. USA) and AG1-X8 (Bio-Rad Laboratories, CA. USA) resins. Sr isotope ratios were measured on a Thermo-Finnegan Triton TIMS at the Laboratoire Magmas et Volcans, Clermont-Ferrand, France. The Sr samples were taken up in 1 M H_3_PO_4_ mixed with tantalum salt as an activator, loaded onto single Re filaments that were previously outgassed at 4.0 ampere (A) for 30 min and then dried down slowly at 1 A. The filaments were then heated up to a temperature of 1400 to1500°C, until a high enough ion beam was reached. Measurements were made in multidynamic mode with two cycles, ion beams being shifted one collector down during the 2^nd^ cycle and samples run until the signal started to drop off in order to maximise internal error. Instrument mass bias was corrected for using a ^86^Sr/^88^Sr ratio of 0.1194 and an exponential mass fractionation law. The accuracy of the ^87^Sr/^86^Sr data was assessed by repeated analyses of ≈30 ng Sr from BHVO-2, which reflected the amount of Sr available for analysis from each moth. This gave an average value of 0.703508±0.000035 (2SD, *n* = 4). The average internal precision of all moth ^87^Sr/^86^Sr analyses was 0.000143 (2SE). Pb isotope ratios were determined with a Nu Instruments MC ICPMS at Victoria University of Wellington. The Pb samples, dissolved in 0.5 wt% Seastar HNO_3_, were introduced to the MS via a DSN-100 desolvating nebulizer (Table S3). Data was acquired using two blocks of 25 integrations of 5 seconds each. NBS 981 calibration standards bracketed each three samples. Repeated analysis of ≈4 ng Pb (to match the average moth sample Pb abundance) JB-2 rock standard gave an average of 2.08718±0.00012 (2SD) for ^208^Pb/^206^Pb and 0.848593±0.000056 for ^207^Pb/^206^Pb (n = 7). The average internal precisions for the actual moth sample Pb isotopes analyses were ±0.00098 2SE for ^208^Pb/^206^Pb and ±0.00049 for ^207^Pb/^206^Pb. Total procedural Pb blanks in this study yielded <15 pg Pb, which represents <0.55% of the average moth sample Pb abundance and required an insignificant blank correction, given the internal precision of the analyses.

All insects were subjected to H isotope analyses. However, logistical constraints necessitated that just six moth samples per region per year were processed for the other biogeochemical markers. Further, due to analytical error, trace element results for the 2008 season was acquired for only four moths from MC, AK, NSW and QLD and two for BP. The markers obtained from the 2008 samples were δ^2^H, ^207^Pb/^206^Pb and ^208^Pb/^206^Pb and elemental concentrations for Li, Al, Sc, Cr, Mn, Ni, Zn, Ga, As, Rb, Sr, Cd, Cs, Ba, W and Pb. To improve the discrimination between the regions, the selection of variables was refined for the 2009 material, and Li, Al, Ca, Sc, Ti, Cr, Co, Ni, Cu, Zn, As, Rb, Sr, Cd, Cs, Ba, La, Ce, W and Pb concentrations were obtained, as well as δ^2^H, ^87^Sr/^86^Sr and ^207^Pb/^206^Pb and ^208^Pb/^206^Pb.

### Statistical analyses

The multivariate datasets from the moth bodies were assessed for regional discrimination for both the 2008 and 2009 data sets. It was necessary to use non-parametric methods, as experimental constraints resulted in fewer samples than the number of variables. Furthermore, with parametric methods, statistical assumptions regarding normally distributed data in multivariate space would have been potentially violated [49] and outlying data points may have led to over-emphasised groups [50]. As such, the datasets were assessed for overall regional difference using PERMANOVA+ (version 1.0.3) (PRIMER-E version 6.1.13) permutational multivariate analysis of variance main test (i.e., overall Pseudo-F); and differences between the individual regions were evaluated using post-hoc PERMANOVA pair-wise tests. The data were log (x+1) transformed, normalised and the analyses carried out using a Euclidean distance resemblance matrix. Both tests used 9999 permutations. The moth multivariate datasets were then assessed for regional grouping and discrimination using a canonical analysis of principal coordinates (PERMANOVA+ CAP analysis).

A dimension reduction process was assessed for the potential to achieve a combination of isotope and trace element values that maximised the separation between the regions by removing non-informative variables. This was accomplished by first ranking the variables according to their relative contribution to the original CAP regional grouping (assessed using the linear correlations between the variables and the CAP ordination axes) for CAP axes 1–3. The least informative variables were eliminated by nominally selecting and discounting those that had a correlation coefficient less than half the largest correlation coefficient on all three CAP axes [51]. The CAP analysis was then re-run with both years' datasets, without the least informative variables. Regional assignment of the moth samples was then tested by ‘Leave-one-out Allocation of Observations to Groups’ cross-validation and re-run pair-wise PERMANOVA tests.

The level of spatial resolution of the multivariate analyses was compared to that of individual variables, after we had identified the most informative individual variables as those having the highest correlaion with the multivariate CAP ordination axes. The regional discrimination potential of these individual variables was assessed using the same cross-validation processes as described above, and further analysed using univariate ANOVA and pair-wise Fishers unrestricted LSD tests (α = 0.05) (GenStat 14.1). Moth δ^2^H sample sizes were uneven at each site and region, and therefore unbalanced ANOVA were employed for this assessment. Regression analyses were also conducted on the un-grouped (i.e., not-mean values) data to test goodness of fit versus latitude. Retrospective power analyses for the δ^2^H, ^87^Sr/^86^Sr, ^207^Pb/^206^Pb (univariate) datasets was also conducted. This was carried out by calculating the differences between the means of the regions and then determining the minimum sample size required for each comparison to be 5% significantly different, using a two-sided, two-sample t-test, with a power of 90% (GenStat 14.1). (The power of a statistical test is defined as the probability that the test will correctly reject the null hypothesis when the null hypothesis is false – i.e. the probability of not committing a Type II error). A standard deviation pooled over all regions was used.

## Results and Discussion

The sample preparation and analytical methodology presented here has enabled the analyses of multiple biogeochemical markers from single insect samples, despite the low concentrations of many of the elements. The regional discrimination potential of the multivariate data was examined initially. This assessment concomitantly identified the most informative individual variables and thus allowed the subsequent comparison of the regional differentiation potential of multivariate vs univariate analyses, which are considered below.

### A multivariate test of provenance differentiation

**Figure 2:**
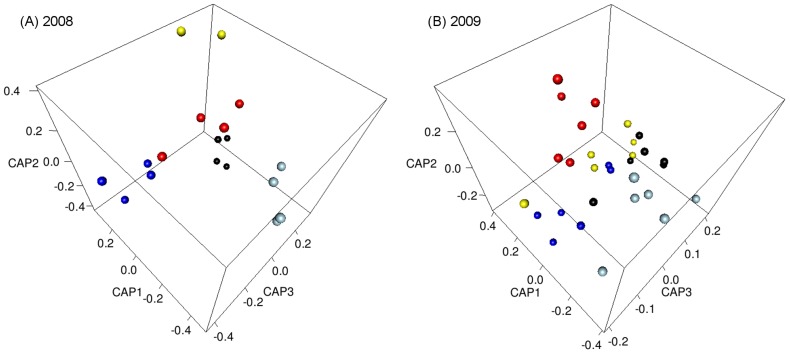
The geographical resolution achieved between the experimental regions using multiple biogeochemical markers. Canonical analysis of principle co-ordinates plots of δ^2^H, trace element concentration (ng/g) and ^206^Pb/^208^Pb, ^207^Pb/^208^Pb data from *H. armigera* adult specimens, reared from Australian and New Zealand sites: (A) March – May 2008 and (B) Jan – March 2009. The 2009 moth data was optimised to remove non-informative variables and also includes ^87^Sr/^86^Sr values. Black  =  MC, yellow  =  BP, dark blue  =  AK, light blue  =  NSW, red  =  QLD.

PERMANOVA analyses identified an overall regional difference for both 2008 and 2009 moth multivariate biogeochemical marker datasets (2008 Pseudo-F_4, 13_ = 2.4051, p(perm)  = 0.0033; 2009 Pseudo-F_4, 25_ = 2.79, p(perm)  = 0.0001). The geographical resolution achieved between individual study areas is illustrated in Figure 2 (animated in Movie S1 & S2) and the associated pair-wise tests (Table S4). In the 2008 dataset, BP moths were not significantly different from moths from any of the other regions, possibly due to the small number of samples from BP in that year (*n* = 2) affecting the comparison with the other regions. The NSW and QLD 2008 moths were also not significantly different from each other. However, the AK and MC moths were distinguishable both from the Australian moths and each other. In contrast, for the 2009 moth dataset, the pair-wise regional comparisons were all significantly different, except for the BP versus MC, and BP versus AK comparisons.

**Figure 3:**
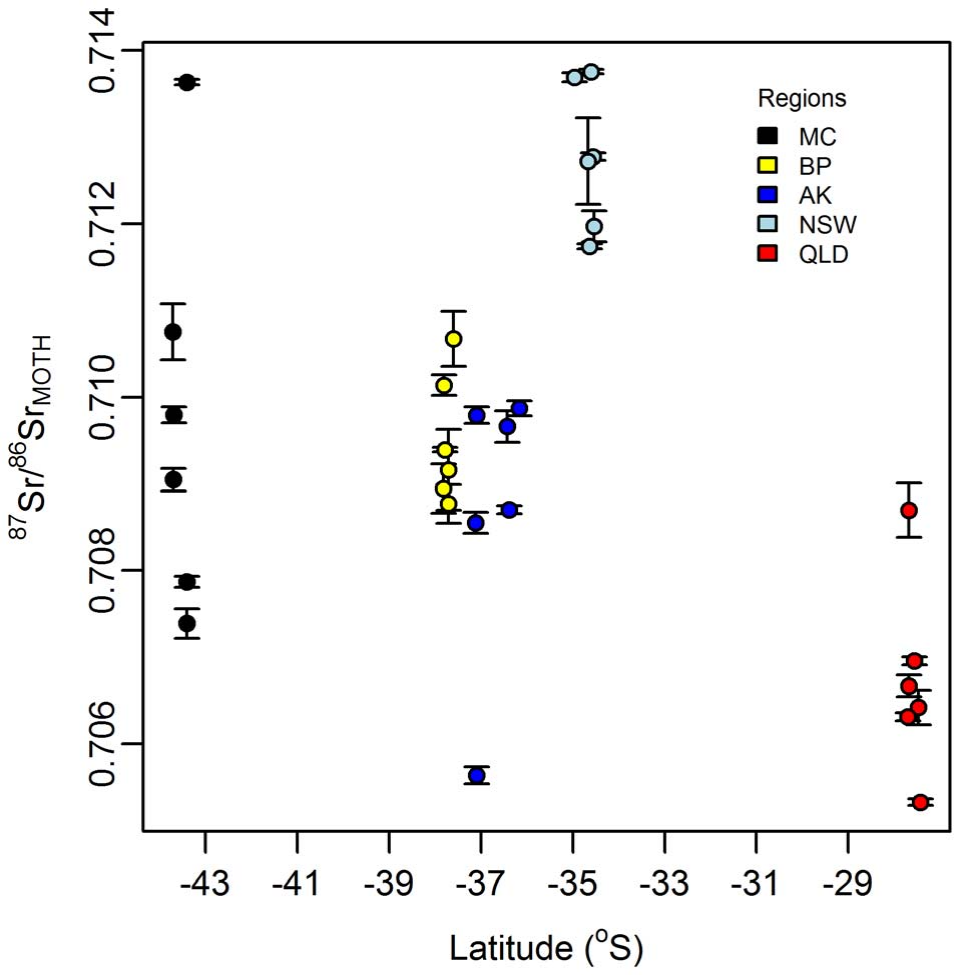
*H. armigera* ^87^Sr/^86^Sr distribution, relative to degress latitude south. Error bars  =  analytical 2SD.

The more powerful geographical separation in the 2009 dataset was primarily due to the addition of ^87^Sr/^86^Sr data. This marker provided robust separation of the moths from the two Australian regions, as well as a lesser but still significant difference between the Australian and New Zealand regions (Figure 3). In addition, the 2009 dataset incorporated a greater number of informative trace elements, including Co, Ce and La, all of which contributed to the improved regional separation (Table S5).

The CAP regional assignment cross-validation tests gave misclassification errors of 22.2% with the 2008 dataset and of 26.7% with the larger 2009 dataset (Table 2). Leaving out the least informative markers (i.e., dimension reduction) was beneficial with the 2009 regional comparison, with the misclassification error being reduced from 36.7%. In contrast, attempts at ‘optimisation’ with the 2008 data in this way increased misclassification error. This indicates that successful provenance determination requires a balanced appraisal of all available markers. It is necessary to consider the potential for the signal to be confounded by biological processes, the degree of overlap between the potential source regions, and the variation within the regions [12], [52].

**Table 2:** Validation tests of regional assignment for individual *H. armigera* samples.

			Classified						
			MC	BP	AK	NSW	QLD	Total	%correct
**2008**	**Original Group**	MC	4	0	0	0	0	4	100
		BP	1	0	0	0	1	2	0
		AK	0	0	3	0	1	4	75
		NSW	0	0	0	3	1	4	75
		QLD	0	0	0	0	4	4	100
									
**2009**	**Original Group**	MC	4	1	1	0	0	6	66.667
		BP	1	4	1	0	0	6	66.667
		AK	2	1	3	0	0	6	50
		NSW	0	0	0	6	0	6	100
		QLD	0	1	0	0	5	6	83.333

Tests of CAP generated groupings using ‘Leave-one-out Allocation of Observations to Groups’ method. 2008 scored 14/18 correct, misclassification error  = 22.2%; 2009 scored 22/30 correct, misclassification error  = 26.7%.

A significant finding regarding provenance assignment for biosecurity is that all the moths from the New Zealand regions are distinguished from the Australian moths using the 2009 dataset. As with the regional pair-wise tests above, the superior inter-country allocation achieved with the latter dataset is attributed primarily to the inclusion of ^87^Sr/^86^Sr as a variable. This result, along with the 73.3% cross-validation success rate, suggests that determining whether a suspect sample has originated from its collection point, or not – i.e., in a biosecurity scenario – is more likely to be successful than not. However, 100% accurate re-allocation was achieved in only one in five regions with the 2009 dataset and two out of five regions with the smaller 2008 dataset. Misclassification between the regions is attributed to the similarities in the mean values and over-lapping ranges of several of the variables. Hence, single insect samples (*n* = 1) may be difficult to reliably assign to place of origin in such circumstances, although discrimination between regions is expected to be more reliable when the sample-sizes are larger.

The most informative variables in the 2008 dataset were: ^207^Pb/^206^Pb,^208^Pb/^206^Pb, δ^2^H, the elemental ratios Pb/Sr, Rb/Sr, Ba/Sr, and the concentrations of Rb and Sr, and Li, Cr, Ga, Ba and Pb (Table S5). In the 2009 dataset, the most informative variables were: ^87^Sr/^86^Sr, δ^2^H, concentrations of Pb, As, Sr, Ba, Cs, all the elemental ratios considered, Pb isotopes, and the variables with significant correlation to the 3^rd^ CAP ordination axes, Ti, Co, Ni, La and Ce. To understand the impact that these might have on the ability to assign origins, and to test the hypothesis that provenance discrimination using multivariate analyses is superior to univariate analysis, these most informative markers individually are considered below.

### Provenance differentiation using δ^2^H_M_

**Figure 4:**
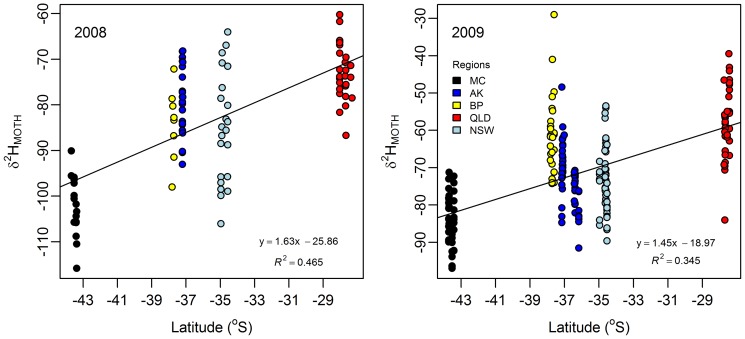
Relationship between *H. armigera* wing δ^2^H values and latitude.

The plot of *H. armigera* wing δ^2^H values (δ^2^H_MOTH_) against latitude confirms a latitudinal continental scale cline in both 2008 and 2009 (Figure 4). The ‘δ^2^H_M_ per degree latitude’ regression is 1.6 and 1.5‰ per degree in the 2008 and 2009 datasets respectively, which is slightly less than the ≈2‰ per degree described by Hobson & Wassenaar et al. [26] for monarch butterflies over eastern North America. Further, the regional δ^2^H_M_ means are significantly different in both years (2008 F_4, 92_ = 33.67; p<0.001; 2009 F_4_,_ 210_ 56.93, p<0.001). However, the δ^2^H_M_ versus latitude R^2^ indicates that at only 46% of the variation was due to latitude for the 2008 dataset, and 35% for the 2009 data. This suggests that biological and/or localized environmental variation within regions is of equal or greater influence than latitude.

Pair-wise comparisons of the δ^2^H_M_ means reveal that, on a population level, the moths from the most southern region, MC, were able to be distinguished from the moths from the more northerly regions, being significantly “lighter” (having lower δ^2^H values) than all the other regions in both years (α = 5%). Beyond this however, δ^2^H_M_ values of the other regions were too similar (Table S6) and/or have too much overlap to be reliably distinguished. The often large sample sizes required to achieve significant differences between the regional δ^2^H_M_ means (calculated retrospectively, Table S7) reiterates the broad scale of spatial resolution and inconsistent individual sample provenance assignment achieved by δ^2^H. Where the δ^2^H_M_ means are distinctly different, there is strong potential for δ^2^H to discriminate moths from different regions. Hence MC can be distinguished from all the other regions by sample sizes of 12 or fewer moths and some comparisons required *n* of only 3 or 4. Conversely, where the δ^2^H_M_ means are close and/or variation is high, the required sample sizes are impractically large, and more than typically collected in biosecurity incursions (commonly 2–6 insects). Further, small sample sizes have high misallocation errors ( =  low power). For example, with *n* = 2 moths, MC, the most distinct region, contrasted to the other regions gave power values ranging from 0.13–0.41 (calculated using a GenStat 14.1, 2-sided, 2-sample t-Test, significance 0.05).

The provenance discrimination achieved with the δ^2^H_M_ univariate analyses for individual samples is compared to that achieved with the multivariate analysis in Table 3. Overall, the total misclassification error for the δ^2^H_M_ univariate analyses was around 55%, which is approximately twice that of the multivariate analysis.

**Table 3:** A comparison of the regional discrimation achieved by multivariate and univariate analyses.

	Original Group	Multivariate assignment	Univariate assignment			
			δ^2^H	δ^2^H	^87^Sr/^86^Sr	^207^Pb/^206^Pb
***n*** ** used for test**		30	30	214	30	30
**% correctly allocated to original group**	MC	66.7%	16.7%	62.8%	33.3%	50%
	BP	66.7%	33.3%	32%	33.3%	0%
	AK	50%	66.7%	39%	33.3%	16.7%
	NSW	100%	50%	29.6%	100%	16.7%
	QLD	83.3%	66.7%	66.7%	83.3%	16.7%
**Total misclassification error**		26.7%	53.3%	55.1%	43.3%	80.0%

The assignement of individual *H. armigera* samples to their original region; generated by CAP ‘Leave-one-out Allocation of Observations to Groups’ method. Uses the 2009 data only; for δ^2^H_MOTH_ both the sub-sample used in the multivariate analysis and full δ^2^H_M_ dataset are given.

This limited geographical resolution is attributed to the large degree of both intra-region and intra-site variation in the δ^2^H moth values. The intra-region δ^2^H_M_ variation spanned 24.8–44.6‰ (for both years), with the average variation being 38‰ in 2008 and 38.9‰ in the 2009 dataset. This is higher than the differences between all the region's means. The intra-site variation comprises the largest component of the within-region variability, being 29.0‰ in the 2008 dataset and 27.8‰ in 2009. This degree of variation is greater than the ≈26‰ (interpolated) intra-region heterogeneity of monarch butterflies [26] and the intra-site variation of ≈28‰ reported in *Inachis io* (Lepidoptera: Nymphalidae) [23]. However, the results herein have similar variability to the intra-site variation that has been observed in *Arhopalus ferus* beetles (Cerambycidae) (up to 31.1‰) [53] and an unidentified insect species (possibly beetle, 40‰) [54]. These results confirm the that quantifying within-population δ^2^H heterogeneity is as necessary for insects as it is for birds [55], [56]. Such within-population heterogeneity needs to be taken into account when using insect δ^2^H information in geographical assignment and is required to propagate error in predictive geographical assignment modelling [57]. It also needs to be taken into account when using insect δ^2^H information in paleoclimate reconstruction, cf. [58].

Furthermore, the relative differences between the regions were inconsistent for the two years, with the δ^2^H_M_ values for the 2009 dataset being significantly “heavier” (having higher δ^2^H values) than the 2008 dataset (F_1,4_ = 27.87, p = 0.006). Although it is important to appreciate that the collections were made at different weeks in each year, the inter-annual variation in δ^2^H_M_ observed indicates that applications using insect δ^2^H need to correct or specifically calibrate the data for each period of interest [59].

While this spatial and temporal heterogeneity of δ^2^H expression makes it difficult to rely upon this as a single marker, it clearly still provides a level of spatial discrimination that can be informative.

### Provenance differentiation using ^87^Sr/^86^Sr_M_

The *H. armigera* ^87^Sr/^86^Sr values (^87^Sr/^86^Sr_M_) from the five regions (Figure 3) were significantly different overall (F_4, 25_ = 14.04, p<0.001). The NSW moths had the highest ^87^Sr/^86^Sr (mean ^87^Sr/^86^Sr  = 0.71278), and QLD the lowest (mean ^87^Sr/^86^Sr  = 0.70673) (a significant difference (α = 5%), Table S8). The New Zealand moth ^87^Sr/^86^Sr values were intermediate to the Australian regions, with all the New Zealand regional means having values of approximately 0.709. Pair-wise comparisons confirmed that the New Zealand moth specimens are significantly different from both NSW and QLD moths (α = 5%). However, the New Zealand regions were not significantly different from each other, with median ^87^Sr/^86^Sr values being separated by only 0.0003.

The capacity of ^87^Sr/^86^Sr data to separate vulnerable biosecurity regions in New Zealand from the relevant risk regions in Australia indicates that, on a population level, Sr isotopes are a potentially powerful tool for provenance determination of intercepted specimens. The minimum sample size required to achieve significant differences between the Australian and New Zealand regions with ^87^Sr/^86^Sr alone was 12 or fewer insects (Table S9). However, the power associated with sample sizes *n* = 2 (a realistic interception sample size) for AK, the highest biosecurity risk centre in New Zealand, versus NSW and QLD is only 0.46 and 0.14 respectively. Further, the New Zealand moths were not able to be assigned to region using ^87^Sr/^86^Sr without impractically large sample sizes. Correspondingly, the total error when using ^87^Sr/^86^Sr_M_ in the univariate reassignment test was 43.3% of individual moths misclassified, as compared to 26.7% misclassification error in the multivariate test (Table 3). Therefore, the regional discrimination potential of strontium isotopes as a single variable cannot be assumed, even for places that are geologically distinct and geographically widely separated.

A prominent characteristic of the ^87^Sr/^86^Sr_M_ data is the within region heterogeneity. MC had the most diverse range, possibly reflecting the geological heterogeneity of the alluvial flood plain. Values varied from 0.7136, which is similar to both Canterbury's rhyolite volcanic [60] and metasiltstone metamorphic rocks [61], to 0.7074 which is consistent with the values reported for Miocene volcanic rocks on the adjacent Banks Peninsular [62]. The AK ^87^Sr/^86^Sr_M_ heterogeneity is also consistent with the geological diversity of the region; a single “low” value (0.7056) lying within the range of values reported for nearby greywacke [63] and the other five values from divergent parts of the Auckland isthmus clustered around 0.7091, possibly reflecting metapelite metamorphic rocks [63] and/or input from marine aerosols [64] (both around 0.709). With regard to the QLD, no geographically close rock or soil ^87^Sr/^86^Sr values have been found in the literature, although the ^87^Sr/^86^Sr_M_ from this region may reflect local trachyte (approximately 0.706) or rhyolite rocks (0.7077) [65]. BP and NSW had the lowest ^87^Sr/^86^Sr dispersion, with ranges of 0.0019 and 0.002 respectively. The degree of within-population ^87^Sr/^86^Sr variation found in the *H. armigera* populations is consistent with that reported in other terrestrial ecology references. For example, within population ^87^Sr/^86^Sr ranges up to 0.0018 have been observed in black-throated blue warblers (but *n* only 2) [66], 0.0025 in snail (Pulmonata, family not given) populations [67] and 1SD values up to 0.00113 reported in tree swallow [68]. In species of Geometridae and Notodontidae (Lepidoptera, species not given) caterpillars at single forest sites, Blum et al. [69] found a ^87^Sr/^86^Sr range of 0.00252, and Blum et al. [70] 0.00307, which are both similar to the within-site ^87^Sr/^86^Sr variance shown here for *H. armigera* (0.00039–0.00203).

As with δ^2^H, therefore, insect ^87^Sr/^86^Sr is also very heterogeneous and has limited utility as a single marker for provenancing. However, the geologically linked expression observed in ^87^Sr/^86^Sr_M_, along with its contribution to the regional differentiation achieved in the multivariate test above, indicates that a combination of geological and climate markers can provide confident regional provenance assignment.

### Provenance differentiation using Pb isotope ratios

**Figure 5:**
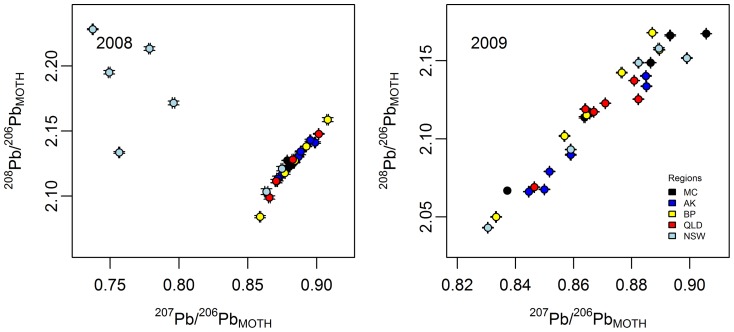
Pb isotope scatter plots from *H. armigera*. Error bars  =  analytical 2SD. Note: the axes for the 2008 dataset is larger scale than 2009.

In the 2008 data, there was significant overall difference between the regional ^207^Pb/^206^Pb_M_ means (F_4, 23_ = 9.94, p = 0.000), but not for ^208^Pb/^206^Pb_M_ (F_4, 23_ = 1.80, p = 0.163), although a pairwise comparison of the 2008 means revealed that NSW ^208^Pb/^206^Pb_M_ was significantly different to all other regions (α = 5%) (Figure 5). Five out of the seven 2008 NSW moths had Pb isotope ratios very significantly shifted from the expected NSW mixing line (^207^Pb/^206^Pb approximately 0.895, ^208^Pb/^206^Pb 2.148), to an ‘exotic value group’ cluster with the median values of ^207^Pb/^206^Pb 0.757 and ^208^Pb/^206^Pb 2.195. No site bias was detected, with the exotic value group being from sites evenly spread over the entire NSW collection region (over a distance of approximately 100km i.e., Ganmain to Coleambally, NSW) and one site yielded both exotic value and non- exotic value samples. To verify that these exotic values were not the result of systematic error, another pair of 2008 NSW moths were subject to separate analytical preparation run and mass spectrometry. These had similar exotic and non-exotic values, which confirm the validity of the earlier analyses. It appears that the affected moths have acquired Pb from sources in addition to the host plant, as their Pb isotope ratios are comprehensively different to that of the associated soils and host plants (average ^207^Pb/^206^Pb_SOIL_ 0.835, ^208^Pb/^206^Pb_SOIL_ 2.073; ^207^Pb/^206^Pb _PLANT_ 0.895, ^208^Pb/^206^Pb_PLANT_ 2.148). The additional source path may be respiratory inhalation, with the exotic Pb source being aerosols or dust particulates. Invertebrate acquisition of Pb by inhalation and accumulation of low concentration Pb contamination has previously been shown in snails (*Cepaea nemoralis*) [71]. The origin of the exotic signal in the present study is theorised to be particulate dust from within few a hundred kilometres west of the collection area. The *H. armigera* exotic value group described here had ^207^Pb/^206^Pb_M_ values similar to the range known for soils at Lake Frome, central South Australia (^207^Pb/^206^Pb, 0.7720, ^208^Pb/^206^Pb, 2.066) [72] and near Adelaide, South Australia [73]. These locations align with the general pattern of dust storms in this region of Australia moving in a southeast direction [74]. In contrast, there was no significant difference between the regional Pb isotope ratio means in the 2009 data (^207^Pb/^206^Pb_M_ F_4, 25_ = 0.54, p = 0.709; ^208^Pb/^206^Pb_M_ F_4, 25_ = 0.83, p = 0.520).

The moth Pb isotope values for 2008 and 2009 were not statistically different, despite the group of exotic values in the 2008 NSW moth dataset (^207^Pb/^206^Pb F_1, 4_ 0.0, p = 0.949; ^208^Pb/^206^Pb F_1, 4_ 2.79, p = 0.17). This is likely to be a consequence of both years' data being widely dispersed, with the 2009 values clustered centrally within the more scattered 2008 data range (Figure 5).

The CAP regional grouping procedure showed that lead isotopes can provide information regarding geographical origin (Table S5). However, lead isotopes appear to be less informative than δ^2^H and ^87^Sr/^86^Sr (Tables S7 & S9 versus Table S10), and had 80% univariate reassignment miscalculation error, which is more than three times that of the multivariate analysis. On-the-other-hand, this work has shown lead isotope data can be obtained from single insect samples, and that the sensitive fine scale resolution available from lead isotope analyses holds considerable promise for tracing ecological linkages and pollution sources which are hitherto not able to be elucidated in entomological science.

### Provenance differentiation using trace element concentrations

**Figure 6:**
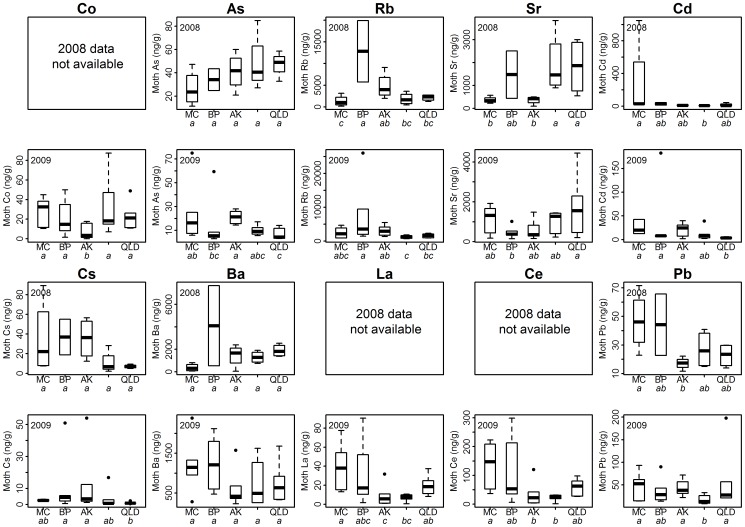
Trace element concentration data for *H. armigera*. Only the most informative elements are shown. Data is displayed as median, quartiles and the minimum/maximum value within 1.5 inter-quartile range; values outside 1.5 IQR are designated by a circle. Regions assigned a different lower case italic letters are significantly different (Fishers unrestricted LSD  = 5%). ‘Data not available’  =  information for that element was not recorded or lost due to analytical error.

The essential elements, which are those linked to common metabolic processes [17], were not geo-location informative, with the elements of atomic number ≤ Arsenic being generally less informative than the elements ≥ atomic number of Rb. Trace element variables that gave the best regional separation across both years are Sr, Cs, Ba and Pb, as well as the Pb/Sr elemental ratio (Table S5). Except for Ba, all of these were univariately significantly different between the regions (Figure 6). However, the values and the relative contributions of the elemental concentrations were not consistent between years. Further, none of the trace elements alone reliably discriminated moths from all of the different geographic regions, as the statistical differences were between only two or three of the five regions. For example, the BP and AK moths had the highest mean Rb and Cs concentrations in both years, and the MC moths the highest Cd values, yet the other regions were not significantly or consistently different (please note however, the results for 2008 BP may not be representative of the entire region, given the n = 2 sample size). The lack of a single geographical trace element marker is consistent with other ecological provenance determination studies, despite the significant differences in regional mean values, e.g., [75]. Nevertheless, elemental concentrations clearly contribute to geographical resolution.

In the 2008 moth trace element data set, the Australian regions had significantly higher Sr concentrations than the AK and MC moths. In contrast, Sr concentrations in the 2009 moth dataset were not significantly different overall (F_4,25_ = 1.69, p = 0.183), although BP and QLD were significantly different from each other in a pair wise test (α = 0.05). The geographical resolution potential of Sr identified here, agrees with the pistachio provenance study of Anderson & Smith [33], with Sr giving the largest source region discrimination potential of all the elements they analysed.

New Zealand moth samples had higher average Cd levels than Australian samples, consistent with studies regarding the elevated levels of Cd in New Zealand agricultural soils [76]. However, despite some regional means being significantly different, moth Cd concentration was not a strong driver in the regional separation CAP analysis. This is due to the large degree of intra-region variation in moth Cd concentration values in all the regions, which results in poor allocation power on an individual moth basis.

Moth average Cs concentrations were also consistently higher in the New Zealand compared to Australian samples, although the statistical distribution of the Cs values may limit the potential of Cs as a biosecurity marker – when sample sizes are typically <6 insects. Most moths had Cs values <10 ng/g, although the larger mean values in both years were skewed by 2–5 moths with Cs values of >50 ng/g. However, all the Cs values >30 ng/g occurred in New Zealand samples and the highest values were most common in BP and AK moths. Thus Cs may be a useful geographical marker for New Zealand with larger sample sizes.

These trace element results are consistent with the avian studies of Norris et al. [77] and Szep et al. [32]. They reported a similar suite of elements (Mg, Cd, Sr, Ba, Rb, Cd, Pb) to be the most informative, and similar degrees of intra-regional heterogeneity – resulting from between site differences (cf. within site variation). This intra-regional variation facilitates better near-distance discrimination than light element stable isotopes, which typically separate populations on continental scales. However, the findings of Torres-Dowdall et al. [78] urge a cautionary interpretation of trace element data. They reported poor re-allocation accuracy for red knot shorebirds (*Calidris canutus*), due to both the lack of trace element marker resolution and because several elemental concentrations, including Sr and Pb, changed as the adult birds aged. The chemical profiles of feathers are believed to be affected by direct absorption from contaminants [79], preening behaviour and chemical leaching [80], [81]. Therefore, although the biochemical processes and age-related changes will be different between birds and insects, as elemental profiles have been shown to also change during the moths' adult stadia [82], trace element profiles from whole moths may not be a reliable indicator of point-of-origin.

## Conclusion

This study is the first evaluation of multiple isotope and trace element markers as a means of insect provenance assignment, as well as the first use of Sr isotopes for this purpose in entomological science. It is also believed to be the first study that has considered Pb isotopic information from insects.

The provenance assignment achieved demonstrates that, with the small samples sizes typical of biosecurity interceptions, none of the biogeochemical markers assessed can individually separate insects reared in different regions of biosecurity importance in New Zealand and Australia. In contrast, a multivariate combination of δ^2^H, ^87^Sr/^86^Sr, ^208^Pb/^206^Pb, ^208^Pb/^207^Pb and selected element concentrations was able to distinguish the region of origin of *H. armigera* for 73.3% of individual moths. This supports the hypothesis that provenance discrimination achievable from multivariate analyses is superior to that of univariate analysis, e.g., [12]. In addition, the value of using multiple independent variables has been highlighted. Specifically, δ^2^H, is a proxy for climate and therefore approximations of latitude, whereas the Sr and Pb isotopic ratios of the moths appear to be primarily that of the source point soils and underlying geology and are independent of climate.

However, it is well recognised that all natural abundance markers have their weaknesses [28]. As such, the advances described above need to be considered in light of various biotic and abiotic limiting factors that are yet to be specifically defined. Identifying and accounting for these limitations is recommended as future research priorities. However, that should not detract from the ongoing use and further development of biogeochemical markers in entomological applications, which could be improved by considering some overarching issues revealed in this study.

Firstly, because of within-region heterogeneity in marker expression there is a strong relationship between confidence of provenance assignment, sample size and the degree of isotopic difference in the potential sources [57]. Similar multifarious marker expression has been observed elsewhere for single or paired isotope systems [67], and needs to be also taken into account in multivariate tracing.

Secondly, the relative discriminating power of the individual variables was inconsistent between the two years that were sampled. In particular, the insect δ^2^H data needs to be calibrated by reference to precipitation δ^2^H data for each period of interest. However, if emphasis is given to those variables that gave significant regional discrimination in both years, as well as those less likely to be affected by inconsistent biological and environmental parameters, which is assumed to include ^87^Sr/^86^Sr [10], temporal discrepancies can likely be minimised.

Lastly, our understanding of how soil and precipitation biogeochemical signals are expressed in insects is limited to the few studies that have actually quantified this relationship. Specifically, such information is available for *H. armigera* [82] and for δ^2^H only, the hoverfly *Episyrphus balteatus* [Diptera] [83], monarch butterflies [26] and several dragon fly species [21]. Therefore, provenance assignment of other insect species currently requires reference populations of the same species from the candidate areas, e.g., [53]. Quantifying these ‘transmission factors’ (e.g., ^2^H fractionation) for a wider range of plant-insect systems will facilitate wider entomological application of this technology in areas such as ecology, forensics and pest management, as well as paleo-climatic reconstruction.

## Supporting Information

Figure S1 **An assessment of the linearity of ICPMS measurement using a dilution series.** Ratios of selected elements' concentrations in diluted solutions (1∶2–1∶4) of an in-house moth body standard over the long term averages of the non-diluted PH-armig moth standard (1∶1). The average distortion on the analytical values, comparing the non-diluted moth standard averages to the most heavily diluted (1∶4) was 3.5%. This indicates that there were minimal matrix effects suffered in the ICP-MS analysis.
(PNG)

Table S1 **ICP-MS instrument settings, conditions and method used for trace element analysis of insect samples.**
(DOCX)

Table S2 **ICPMS trace element measurement precision.** The averages of dilute (10%) calibration standard, in-house moth body standard and NBS 1575 Pine needle external standard from each analytical run. All concentrations are ng/g calculated using sample and dilution weights. %CV  =  coefficient of variation. The average recovery of elements for NBS 1575 is versus the following published values: A =  Certificate of Analysis (Reed, 1993); B =  (Freitas et al., 2008); C =  (Saitoh et al., 2002); D =  (Asfaw & Wibetoe, 2006); E =  (Taylor et al., 2007).
(DOCX)

Table S3 **Typical instrument operating conditions of the Victoria University Nu MC-ICP-MS and the DSN-100 parameters used for Pb isotope analysis.**
(DOCX)

Table S4 **Pair-wise tests of regional differences for** ***H. armigera*** **populations, showing significant differences between the collection regions.** Generated by PERMANOVA analyses of the multivariate datasets (using Euclidean distance resemblance matrices). †  =  p<0.10; *  =  p<0.05; **  =  p<0.01.
(DOCX)

Table S5 **Relative contribution of the individual markers to the CAP regional grouping.** Expressed as Pearson's correlation coefficient (i.e., linear measure of assocaition) between the individual markers (ignoring all others) and the CAP ordination axes within the mulitvariate data cloud. 2-sided significance test expressed as †  = 10%; *  = 5%; **  = 1%.; 2008 data df  = 16, 2009 df  = 18. 2009 data is an optimized suite.
(DOCX)

Table S6 ***H. armigera*** **wing δ^2^H summary table.** Showing regional δ^2^H_M_ averages ± 1SD; values within a row that are followed by a different letter are significantly different (Fishers unrestricted LSD  = 5%).
(DOCX)

Table S7 **Retrospective power analyses for** ***H. armigera*** **δ^2^H.** To detect significant differences between the regional means (Δ‰), at a two-sided significance level of 0.05 with a power of 0.90 using a two-sample t-test, the calculated sample size (n) would be required for each sample. A standard deviation pooled across all regions was used in each power analysis.
(DOCX)

Table S8 ***H. armigera*** **^87^Sr/^86^Sr summary table.** Showing regional ^87^Sr/^86^Sr averages ± 1SD; values within a row that are followed by a different letter are significantly different (Fishers unrestricted LSD  = 5%). *n* = 6 for each region.
(DOCX)

Table S9 **Retrospective power analysis for the** ***H. armigera*** **^87^Sr/^86^Sr data.** To detect significant differences between the regional means (Δ), at a two-sided significance level of 0.05 with a power of 0.90 using a two-sample t-test, replication of the calculated n for each sample is required.
(DOCX)

Table S10 **Retrospective power analysis for the** ***H. armigera*** **^207^Pb/^206^Pb data.** To detect significant differences between the regional means (Δ), at a two-sided significance level of 0.05 with a power of 0.90 using a two-sample t-test, replication of the calculated n for each sample is required.
(DOCX)

Movie S1 **Animation of the geographical resolution achieved between the experimental regions using multiple biogeochemical markers, 2008.** Canonical analysis of principle co-ordinates plots of δ^2^H, trace element concentration (ng/g) and ^206^Pb/^208^Pb, ^207^Pb/^208^Pb data from *H. armigera* adult specimens, reared from Australian and New Zealand sites, March – May 2008. Black  =  MC, yellow  =  BP, dark blue  =  AK, light blue  =  NSW, red  =  QLD.
(MP4)

Movie S2 **Animation of the geographical resolution achieved between the experimental regions using multiple biogeochemical markers, 2009.** Canonical analysis of principle co-ordinates plots of δ^2^H, trace element concentration (ng/g) and ^206^Pb/^208^Pb, ^207^Pb/^208^Pb data from *H. armigera* adult specimens, reared from Australian and New Zealand sites, Jan – March 2009. The 2009 moth data was optimised to remove non-informative variables and also includes ^87^Sr/^86^Sr values. Black  =  MC, yellow  =  BP, dark blue  =  AK, light blue  =  NSW, red  =  QLD.
(MP4)

## References

[1] Goldson SL, Rowarth JS, Caradus JR (2005) The impact of invasive invertebrate pests in pastoral agriculture: a review. New Zealand Journal of Agricultural Research 48: 401–415.

[2] Pimentel D (2011) Biological Invasions: Economic and Environmental Costs of Alien Plant, Animal, and Microbe Species, 2nd Edition. Florida: CRC Press 463 p.

[3] ISSG (2008) IUCN/SSC Invasive Species Specialist Group. ISSG. Available: http://www.issg.org/

[4] FAO (2002) Guidelines for Phytosanitary Certificates. International Standards for Phytosanitary Measures: FAO. Available: http://www.fao.org/DOCREP/004/Y3241E/Y3241E00.HTM

[5] Lavandero B, Wratten S, Hagler J, Jervis M (2004) The need for effective marking and tracking techniques for monitoring the movements of insect predators and parasitoids. International Journal of Pest Management 50: 147–151.

[6] Fitzpatrick BM, Fordyce JA, Niemiller ML, Reynolds RG (2012) What can DNA tell us about biological invasions? Biological Invasions 14: 245–253.

[7] Barr NB (2009) Pathway analysis of *Ceratitis capitata* (Diptera: Tephritidae) using mitochondrial DNA. J Econ Entomol 102: 401–411. 19253662 10.1603/029.102.0153

[8] Manel S, Schwartz MK, Luikart G, Taberlet P (2003) Landscape genetics: combining landscape ecology and population genetics. Trends in Ecology & Evolution 18: 189–197.

[9] Hobson KA, Wassenaar LI (2008) Tracking Animal Migration with Stable Isotopes; Hobson KA, Wassenaar LI, editors. Amsterdam: Academic Press. 160 p.

[10] Capo RC, Stewart BW, Chadwick OA (1998) Strontium isotopes as tracers of ecosystem processes: theory and methods. Geoderma 82: 197–225.

[11] Bowen GJ (2010) Isoscapes: Spatial Pattern in Isotopic Biogeochemistry. Annual Review of Earth and Planetary Sciences 38: 161–187.

[12] Oulhote Y, Le Bot B, Deguen S, Glorennec P (2011) Using and interpreting isotope data for source identification. TrAC Trends in Analytical Chemistry 30: 302–312.

[13] McLean JA, Bennett RB (1978) Characterization of two *Gnathotrichus sulcatus* populations by X-ray energy spectrometry. Environmental Entomology 7: 93–96.

[14] McLean JA, Shepherd RF, Bennett RB (1979) Chemoprinting by X-ray energy spectrometry: We are where we eat. In: Rabb RL, Kennedy GG, editors. Movement of highly mobile insects: concepts and meth[o]dology in research: proceedings of a conference “Movement of selected species of Lepidoptera in the southeastern United States,” April 9–11, 1979. Raleigh, N.C.: Dept. of Entomology, North Carolina State University. pp. 369–379.

[15] Turner RH, Bowden J (1983) X-ray-microanalysis applied to the study of insect migration with special reference to rice bug, *Nilaparvata lugens*. Scanning Electron Microscopy: 873–878.

[16] Bowden J, Brown G, Stride T (1979) The application of X-ray spectrometry to analysis of elemental composition (chemoprinting) in the study of migration of *Noctua pronuba* L. Ecological Entomology 4: 199–204.

[17] Mertz W (1981) The essential trace elements. Science 213: 1332–1338. 7022654 10.1126/science.7022654

[18] Bowden J, Digby PGN, Sherlock PL (1984) Studies of elemental composition as a biological marker in insects .1. The influence of soil type and host-plant on elemental composition of *Noctua-pronuba* (L) (Lepidoptera, Noctuidae). Bulletin of Entomological Research 74: 207–225.

[19] Dempster JP, Lakhani KH, Coward PA (1986) The use of chemical-composition as a population marker in insects - a study of the Brimstone Butterfly. Ecological Entomology 11: 51–65.

[20] Wassenaar LI, Hobson KA (1998) Natal origins of migratory monarch butterflies at wintering colonies in Mexico: New isotopic evidence. Proceedings of the National Academy of Sciences of the United States of America 95: 15436–15439. 9860986 10.1073/pnas.95.26.15436 PMC28060

[21] Hobson KA, Soto DX, Paulson DR, Wassenaar LI, Matthews JH (2012) A dragonfly (δ^2^H) isoscape for North America: a new tool for determining natal origins of migratory aquatic emergent insects. Methods in Ecology and Evolution 3: 766–772.

[22] Abney MR, Sorenson CE, Gould F, Bradley JR (2008) Limitations of stable carbon isotope analysis for determining natal host origins of tobacco budworm, *Heliothis virescens* . Entomologia Experimentalis et Applicata 126: 46–52.

[23] Brattström O, Wassenaar LI, Hobson KA, Åkesson S (2008) Placing butterflies on the map – testing regional geographical resolution of three stable isotopes in Sweden using the monophagus peacock *Inachis io* . Ecography 31: 490–498.

[24] Spence KO, Rosenheim JA (2005) Isotopic enrichment in herbivorous insects: a comparative field-based study of variation. Oecologia 146: 89–97. 16012818 10.1007/s00442-005-0170-9

[25] Lancaster J, Waldron S (2001) Stable isotope values of lotic invertebrates: Sources of variation, experimental design, and statistical interpretation. Limnology and Oceanography 46: 723–730.

[26] Hobson KA, Wassenaar LI, Taylor OR (1999) Stable isotopes (δD and δ^13^C) are geographic indicators of natal origins of monarch butterflies in eastern North America. Oecologia 120: 397–404. 28308016 10.1007/s004420050872

[27] Husheer T, Frew R (2005) Stable Isotope investigation of painted apple moth and fall webworm. IsoTrace New Zealand Limited report to Biosecurity New Zealand: 13 p.

[28] Hobson KA (2005) Using stable isotopes to trace long-distance dispersal in birds and other taxa. Diversity and Distributions 11: 157–164.

[29] Kelly SD, Heaton K, Hoogewerff J (2005) Tracing the geographical origin of food: the application of multi-element and multi-isotope analysis. Trends in Food Science and Technology 16: 555–567.

[30] Rossmann A (2001) Determination of stable isotope ratios in food analysis. Food Reviews International 17: 347–381.

[31] Rubenstein DR, Hobson KA (2004) From birds to butterflies: animal movement patterns and stable isotopes. Trends in Ecology & Evolution 19: 256–263. 16701265 10.1016/j.tree.2004.03.017

[32] Szep T, Moller AP, Vallner J, Kovacs B, Norman D (2003) Use of trace elements in feathers of sand martin *Riparia riparia* for identifying moulting areas. Journal of Avian Biology 34: 307–320.

[33] Anderson KA, Smith BW (2005) Use of chemical profiling to differentiate geographic growing origin of raw pistachios. Journal of Agricultural and Food Chemistry 53: 410–418. 15656681 10.1021/jf048907u

[34] Kelly SD, Baxter M, Chapman S, Rhodes C, Dennis J, et al (2002) The application of isotopic and elemental analysis to determine the geographical origin of premium long grain rice. European Food Research and Technology 214: 72–78.

[35] Fitt GP (1989) The Ecology of *Heliothis* Species in Relation to Agroecosystems. Annual Review of Entomology 34: 17–53.

[36] Feng H, Gould F, Huang Y, Jiang Y, Wu K (2010) Modeling the population dynamics of cotton bollworm *Helicoverpa armigera* (Hubner) (Lepidoptera: Noctuidae) over a wide area in northern China. Ecological Modelling 221: 1819–1830.

[37] Brevault T, Achaleke J, Sougnabe SP, Vaissayre M (2008) Tracking pyrethroid resistance in the polyphagous bollworm, *Helicoverpa armigera* (Lepidoptera: Noctuidae), in the shifting landscape of a cotton-growing area. Bulletin of Entomological Research 98: 565–573. 18590595 10.1017/S0007485308005877

[38] Biosecurity-New-Zealand (2006) Pathway Analysis Report 2005-06. Ministry of Agriculture and Forestry. BMG 05-06/12. 51 p.

[39] Biosecurity-New-Zealand (2011) Pathway Analysis Report 2010-11. Ministry of Agriculture and Forestry. BMG 10-11/12.

[40] Common IFB (1953) The Australian Species of *Heliothis* (Lepidoptera: Noctuidae) and their Pest Status. Australian Journal of Zoology 1: 319–344.

[41] Pogue MG (2004) A new synonym of *Helicoverpa zea* (Boddie) and differentiation of adult males of *H. zea* and *H. armigera* (Hübner) (Lepidoptera: Noctuidae: Heliothinae). Annals of the Entomological Society of America 97: 1222–1226.

[42] Armstrong KF, Ball SL (2005) DNA barcodes for biosecurity: invasive species identification. Philosophical Transactions of the Royal Society B-Biological Sciences 360: 1813–1823. 10.1098/rstb.2005.1713 PMC1609225 16214740

[43] Sauer PE, Schimmelmann A, Sessions AL, Topalov K (2009) Simplified batch equilibration for D/H determination of non-exchangeable hydrogen in solid organic material. Rapid Communications in Mass Spectrometry 23: 949–956. 19241415 10.1002/rcm.3954

[44] Schimmelmann A, Lewan MD, Wintsch RP (1999) D/H isotope ratios of kerogen, bitumen, oil, and water in hydrous pyrolysis of source rocks containing kerogen types I, II, IIS, and III. Geochimica et Cosmochimica Acta 63: 3751–3766.

[45] Wassenaar LI, Hobson KA (2010) Two new keratin standards (δ^2^H, δ^18^O) for daily laboratory use in wildlife and forensic isotopic studies. The 7th International Conference on Applications of Stable Isotope Techniques to Ecological Studies. University of Alaska, Fairbanks, Alaska, USA.

[46] Font L, Nowell GM, Graham Pearson D, Ottley CJ, Willis SG (2007) Sr isotope analysis of bird feathers by TIMS: a tool to trace bird migration paths and breeding sites. Journal of Analytical Atomic Spectrometry 22: 513–522.

[47] Pin C, Bassin C (1992) Evaluation of a strontium-specific extraction chromatographic method for isotopic analysis in geological materials. Analytica Chimica Acta 269: 249–255.

[48] Baker J, Peate D, Waight T, Meyzen C (2004) Pb isotopic analysis of standards and samples using a ^207^Pb-^204^Pb double spike and thallium to correct for mass bias with a double-focusing MC-ICP-MS. Chemical Geology 211: 275–303.

[49] Baxter MJ (2008) Mathematics, statistics and archaeometry: The past 50 years or so*. Archaeometry 50: 968–982.

[50] StatSoft_Inc (2011) Electronic Statistics Textbook. In: Hill T, Lewicki P, editors. STATISTICS: Methods and Applications. Tulsa,OK, USA: StatSoft.

[51] Timm NH (2002) Applied Multivariate analysis. New York: Springer-Verlag.

[52] Montgomery J, Evans JA, Cooper RE (2007) Resolving archaeological populations with Sr-isotope mixing models. Applied Geochemistry 22: 1502–1514.

[53] Holder PW (2013) *Arhopalus ferus* (Cerambycidae) hydrogen isotope data as a place of origin marker. Available: http://dx.doi.org/10.6084/m9.figshare.813315

[54] Schimmelmann A, Miller RF, Leavitt SW (1993) Hydrogen isotopic exchange and stable isotope ratios in cellulose, wood, chitin, and amino compounds. In: Swart PK, Lohmann KC, McKenzie J, Savin S, editors. Climate Change in Continental Isotopic Records. Washington, DC: American Geophysical Union. pp. 367–374.

[55] Wassenaar LI, Hobson KA (2006) Stable-hydrogen isotope heterogeneity in keratinous materials: mass spectrometry and migratory wildlife tissue subsampling strategies. Rapid Communications in Mass Spectrometry 20: 2505–2510. 16862621 10.1002/rcm.2626

[56] Langin K, Reudink M, Marra P, Norris D, Kyser T, et al (2007) Hydrogen isotopic variation in migratory bird tissues of known origin: implications for geographic assignment. Oecologia 152: 449–457. 17370093 10.1007/s00442-007-0669-3

[57] Wunder MB, Norris DR (2008) Improved estimates of certainty in stable-isotope-based methods for tracking migratory animals. Ecological Applications 18: 549–559. 18488615 10.1890/07-0058.1

[58] Gröcke DR, Hardenbroek M, Sauer PE, Elias SA (2011) Hydrogen Isotopes in Beetle Chitin. In: Gupta NS, editor. Chitin: Springer Netherlands. pp. 105–116.

[59] Farmer A, Cade B, Torres-Dowdall J (2008) Fundamental limits to the accuracy of deuterium isotopes for identifying the spatial origin of migratory animals. Oecologia 158: 183–192. 18810500 10.1007/s00442-008-1143-6

[60] Barley ME (1987) Origin and evolution of mid-cretaceous, garnet-bearing, intermediate and silicic volcanics from Canterbury, New Zealand. Journal of Volcanology and Geothermal Research 32: 247–267.

[61] Adams CJ, Maas R (2004) Rb-Sr age and strontium isotopic characterisation of the Torlesse Supergroup in Canterbury, New Zealand, and implications for the status of the Rakaia Terrane. New Zealand Journal of Geology and Geophysics 47: 201–217.

[62] Timm C, Hoernle K, Van Den Bogaard P, Bindeman I, Weaver S (2009) Geochemical Evolution of Intraplate Volcanism at Banks Peninsula, New Zealand: Interaction Between Asthenospheric and Lithospheric Melts. Journal of Petrology 50: 989–1023.

[63] Adams CJ, Maas R (2004) Age/isotopic characterisation of the Waipapa Group in Northland and Auckland, New Zealand, and implications for the status of the Waipapa Terrane. New Zealand Journal of Geology and Geophysics 47: 173–187.

[64] Faure G, Mensing TM (2005) Isotopes: Principles and Applications. Hoboken, N.J.: Wiley. 897 p.

[65] GeoRoc (2012) Geochemistry of Rocks of the Oceans and Continents. In: Sarbas B, editor. Mainz Germany. Available: http://georoc.mpch-mainz.gwdg.de/georoc/

[66] Chamberlain CP, Blum JD, Holmes RT, Feng X, Sherry TW, et al (1997) The use of isotope tracers for identifying populations of migratory birds. Oecologia 109: 132–141. 10.1007/s004420050067 28307603

[67] Frei KM, Frei R (2011) The geographic distribution of strontium isotopes in Danish surface waters - A base for provenance studies in archaeology, hydrology and agriculture. Applied Geochemistry 26: 325–340.

[68] Sellick MJ, Kyser TK, Wunder MB, Chipley D, Norris DR (2009) Geographic Variation of Strontium and Hydrogen Isotopes in Avian Tissue: Implications for Tracking Migration and Dispersal. PLoS ONE 4: e4735. 19266102 10.1371/journal.pone.0004735 PMC2649426

[69] Blum JD, Taliaferro EH, Weisse MT, Holmes RT (2000) Changes in Sr/Ca, Ba/Ca and ^87^Sr/^86^Sr ratios between trophic levels in two forest ecosystems in the northeastern U.S.A. Biogeochemistry 49: 87–101.

[70] Blum JD, Taliaferro EH, Holmes RT (2001) Determining the sources of calcium for migratory songbirds using stable strontium isotopes. Oecologia 126: 569–574. 28547242 10.1007/s004420000550

[71] Notten MJM, Walraven N, Beets CJ, Vroon P, Rozema J, et al (2008) Investigating the origin of Pb pollution in a terrestrial soil-plant-snail food chain by means of Pb isotope ratios. Applied Geochemistry 23: 1581–1593.

[72] Kamber BS, Marx SK, McGowan HA (2010) Comment on “Lead isotopic evidence for an Australian source of Aeolian dust to Antarctica at times over the last 170,000 years” by P. De Deckker, M. Norman, I.D. Goodwin, A. Wain and F.X. Gingele Palaeogeography, Palaeoclimatology, Palaeoecology 285 (2010)205-223. Palaeogeography Palaeoclimatology Palaeoecology 298: 432–436.

[73] Gulson BL, Tiller KG, Mizon KJ, Merry RH (1981) Use of lead isotopes in soils to identify the source of lead contamination near Adelaide, South Australia. Environmental Science & Technology 15: 691–696. 22299747 10.1021/es00088a008

[74] Revel-Rolland M, De Deckker P, Delmonte B, Hesse PP, Magee JW, et al (2006) Eastern Australia: A possible source of dust in East Antarctica interglacial ice. Earth and Planetary Science Letters 249: 1–13.

[75] Kaimal B, Johnson R, Hannigan R (2009) Distinguishing breeding populations of mallards (*Anas platyrhynchos*) using trace elements. Journal of Geochemical Exploration 102: 176–180.

[76] Longhurst RD, Roberts AHC, Waller JE (2004) Concentrations of arsenic, cadmium, copper, lead, and zinc in New Zealand pastoral topsoils and herbage. New Zealand Journal of Agricultural Research 47: 23–32.

[77] Norris DR, Lank D, Pither J, Chipley D, Ydenberg R, et al (2007) Trace element profiles as unique identifiers of western sandpiper (*Calidris maur*i) populations. Canadian Journal of Zoology 85: 579–583.

[78] Torres-Dowdall J, Farmer AH, Abril M, Bucher EH, Ridley I (2010) Trace Elements Have Limited Utility for Studying Migratory Connectivity in Shorebirds that Winter in Argentina. The Condor 112: 490–498.

[79] Goede AA, Debruin M (1986) The use of bird feathers for indicating heavy-metal pollution. Environmental Monitoring and Assessment 7: 249–256. 24253671 10.1007/BF00418017

[80] Edwards WR, Smith KE (1984) Exploratory experiments on the stability of mineral profiles of feathers. Journal of Wildlife Management 48: 853–866.

[81] Bortolotti GR, Szuba KJ, Naylor BJ, Bendell JF (1988) Stability of mineral profiles of spruce grouse feathers. Journal of Wildlife Management 52: 736–743.

[82] Holder PW (2013) Isotopes and trace elements as geographic origin markers for biosecurity pests. A thesis submitted in partial fulfilmentof the requirements for the Degree of Doctor of Philosophy: Lincoln University.

[83] Ouin A, Menozzi P, Coulon M, Hamilton AJ, Sarthou JP, et al (2011) Can deuterium stable isotope values be used to assign the geographic origin of an auxiliary hoverfly in south-western France? Rapid Communications in Mass Spectrometry 25: 2793–2798. 21913257 10.1002/rcm.5127

