# Supplementary figures and images for: Isotopes and Trace Elements as Natal Origin Markers of Helicoverpa armigera – An Experimental Model for Biosecurity Pests
Source: PLoS One. 2014 Mar 24;9(3):e92384. doi: 10.1371/journal.pone.0092384 (PMC3963883; doi:10.1371/journal.pone.0092384)

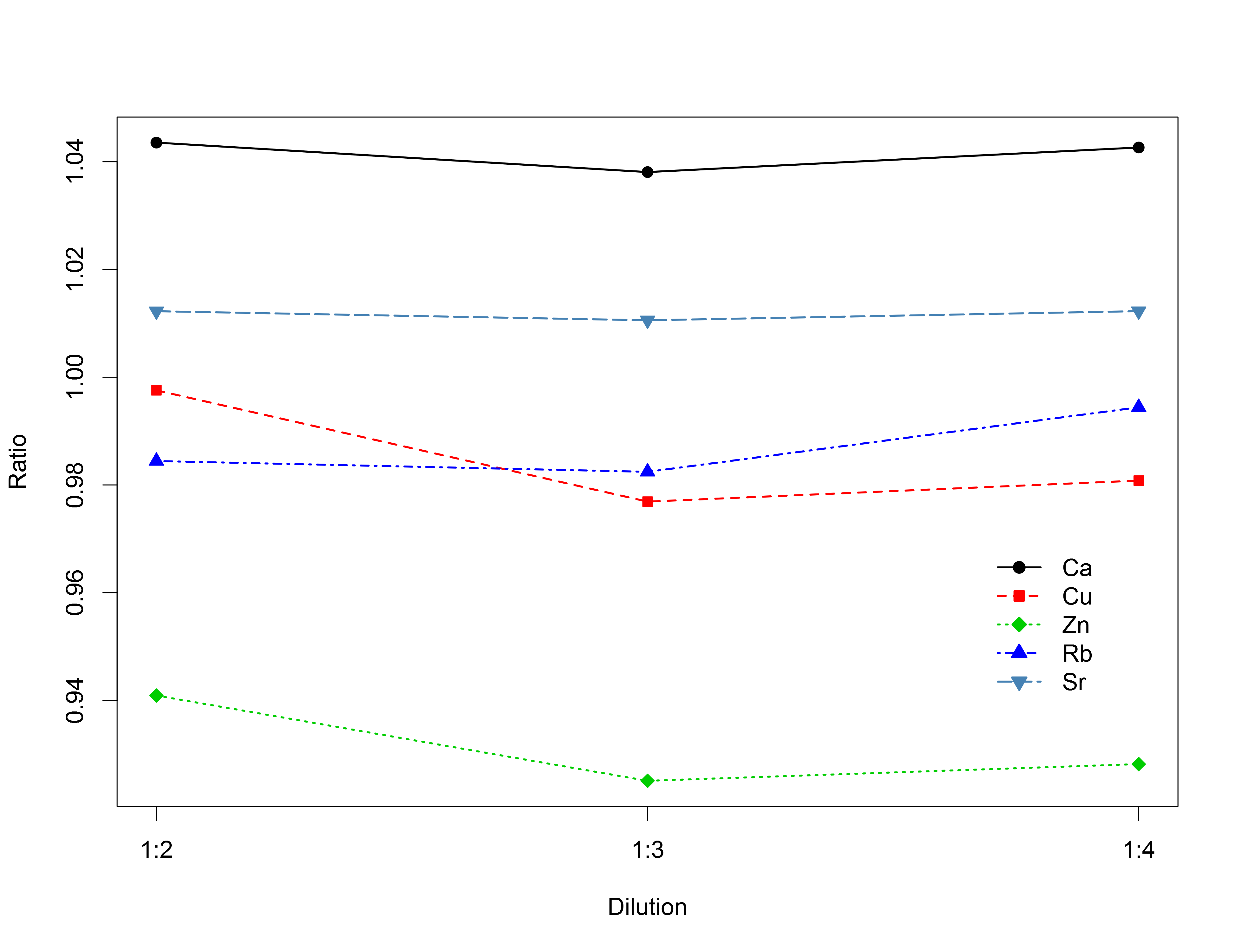

Supplement: Figure S1 — An assessment of the linearity of ICPMS measurement using a dilution series. Ratios of selected elements' concentrations in diluted solutions (1∶2–1∶4) of an in-house moth body standard over the long term averages of the non-diluted PH-armig moth standard (1∶1). The average distortion on the analytical values, comparing the non-diluted moth standard averages to the most heavily diluted (1∶4) was 3.5%. This indicates that there were minimal matrix effects suffered in the ICP-MS analysis. (PNG) [file pone.0092384.s001.png]
