# Supplementary material for: Isotopes and Trace Elements as Natal Origin Markers of Helicoverpa armigera – An Experimental Model for Biosecurity Pests
Source: PLoS One. 2014 Mar 24;9(3):e92384. doi: 10.1371/journal.pone.0092384 (PMC3963883; doi:10.1371/journal.pone.0092384)
Supplement: Table S1 — ICP-MS instrument settings, conditions and method used for trace element analysis of insect samples. (DOCX) [file pone.0092384.s002.docx]

| **ICP-MS system** | Agilent 7500CS octopole |
| --- | --- |
| **Acquisition mode** | Peak hopping |
| **RF power** | 1400 – 1450 W |
| **RF matching** | 1.75 – 1.79 V |
| **Carrier gas (Ar) flow rate** | 0.70 – 0.75 L/min. |
| **Reaction Cell** | Not activated |
| **Nebuliser pump** | 0.14rps |
| **Background acquisition** | 90 s |
| **Sample/standard acquisition** | 180 s |
| **Washout time** | 210 s |
| **Isotopes measured and integration times** | 10 ms: ^7^Li, ^9^Be, ^27^Al, ^43^Ca, ^45^Sc, ^47^Ti, ^49^Ti, ^53^Cr, ^59^Co, ^60^Ni, ^63^Cu, ^65^Cu, ^67^Zn, ^75^As, ^77^Se, ^88^Sr |
|  | 20 ms: ^85^Rb, ^111^Cd, ^133^Cs, ^137^Ba, ^139^La, ^140^Ce, ^182^W, ^205^Tl |
|  | 50 ms: ^206^Pb, ^208^Pb |

**Table S1.** **ICP-MS instrument settings, conditions and method used for trace element analysis of insect samples**.
