# Supplementary material for: Isotopes and Trace Elements as Natal Origin Markers of Helicoverpa armigera – An Experimental Model for Biosecurity Pests
Source: PLoS One. 2014 Mar 24;9(3):e92384. doi: 10.1371/journal.pone.0092384 (PMC3963883; doi:10.1371/journal.pone.0092384)
Supplement: Table S2 — ICPMS trace element measurement precision. The averages of dilute (10%) calibration standard, in-house moth body standard and NBS 1575 Pine needle external standard from each analytical run. All concentrations are ng/g calculated using sample and dilution weights. %CV = coefficient of variation. The average recovery of elements for NBS 1575 is versus the following published values: A = Certificate of Analysis (Reed, 1993); B = (Freitas et al., 2008); C = (Saitoh et al., 2002); D = (Asfaw & Wibetoe, 2006); E = (Taylor et al., 2007). (DOCX) [file pone.0092384.s003.docx]

**Table S2** **ICPMS trace element measurement precision**. The averages of dilute (10%) calibration standard, in-house moth body standard and NBS 1575 Pine needle external standard from each analytical run. All concentrations are ng/g calculated using sample and dilution weights. %CV = coefficient of variation. The average recovery of elements for NBS 1575 is versus the following published values: A = Certificate of Analysis ([Reed, 1993](#_ENREF_3)); B = ([Freitas et al., 2008](#_ENREF_2)); C = ([Saitoh et al., 2002](#_ENREF_4)); D = ([Asfaw & Wibetoe, 2006](#_ENREF_1)); E = ([Taylor et al., 2007](#_ENREF_5)).

| **Dilute Synthetic Std** | | | | | | | | | | | | | | | | | | | | | | | | | | | | | | | | | | | | | | | | | | | | | | | | | | | | | | | | | | | | | | | | | | | | | | | | | | | | | |
| --- | --- | --- | --- | --- | --- | --- | --- | --- | --- | --- | --- | --- | --- | --- | --- | --- | --- | --- | --- | --- | --- | --- | --- | --- | --- | --- | --- | --- | --- | --- | --- | --- | --- | --- | --- | --- | --- | --- | --- | --- | --- | --- | --- | --- | --- | --- | --- | --- | --- | --- | --- | --- | --- | --- | --- | --- | --- | --- | --- | --- | --- | --- | --- | --- | --- | --- | --- | --- | --- | --- | --- | --- | --- | --- | --- | --- | --- |
|  | | **^7^Li** | | **^9^Be** | | | | | **^27^Al** | | | **^43^Ca** | | **^45^Sc** | | | | **^47^Ti** | | **^49^Ti** | | | | **^53^Cr** | | **^59^Co** | | | **^60^Ni** | | | **^63^Cu** | | | **^65^Cu** | | | **^67^Zn** | | | **^75^As** | | | **^77^Se** | | | **^85^Rb** | | | **^88^Sr** | | | **^111^Cd** | | | **^133^Cs** | | | **^137^Ba** | | | **^139^La** | | | **^140^Ce** | | | **^182^W** | | **^183^W** | | **^205^Tl** | | **^206^Pb** | | **^208^Pb** | |
| **Mean** | | 1 | | 11 | | | | | 108 | | | 5240 | | 10 | | | | 523 | | 525 | | | | 11 | | 11 | | | 11 | | | 11 | | | 11 | | | 11 | | | 1 | | | 1 | | | 11 | | | 11 | | | 10 | | | 1 | | | 11 | | | 1 | | | 1 | | | 10 | | 11 | | 11 | | 11 | | 11 | |
| **SD** | | 0 | | 0 | | | | | 4 | | | 146 | | 1 | | | | 10 | | 9 | | | | 0 | | 0 | | | 0 | | | 0 | | | 0 | | | 2 | | | 0 | | | 0 | | | 0 | | | 0 | | | 0 | | | 0 | | | 0 | | | 0 | | | 0 | | | 0 | | 0 | | 0 | | 0 | | 0 | |
| **%CV** | | 3 | | 2 | | | | | 3 | | | 3 | | 12 | | | | 2 | | 2 | | | | 2 | | 2 | | | 2 | | | 3 | | | 2 | | | 17 | | | 3 | | | 29 | | | 3 | | | 2 | | | 2 | | | 2 | | | 1 | | | 2 | | | 2 | | | 2 | | 2 | | 6 | | 1 | | 3 | |
| **NBS-SRM 1575 Pine Needle** | | | | | | | | | | | | | | | | | | | | | | | | | | | | | | | | | | | | | | | | | | | | | | | | | | | | | | | | | | | | | | | | | | | | | | | | | | | | | |
|  | | | **^7^Li** | | | **^9^Be** | | **^27^Al** | | **^43^Ca** | | | **^45^Sc** | | | **^47^Ti** | | | **^49^Ti** | | | **^53^Cr** | | | **^59^Co** | | | **^60^Ni** | | | **^63^Cu** | | | **^65^Cu** | | | **^67^Zn** | | **^75^As** | | | | **^77^Se** | | | **^85^Rb** | | | **^88^Sr** | | | **^111^Cd** | | | **^133^Cs** | | | **^137^Ba** | | | **^139^La** | | | **^140^Ce** | | | **^182^W** | | | **^183^W** | | **^205^Tl** | | **^206^Pb** | | **^208^Pb** | |
| **Mean** | | | 108 | | | 7 | |  | | 3808257 | | | 57 | | | 5249 | | | 4177 | | | 2050 | | | 93 | | | 2064 | | | 2612 | | | 2612 | | | 52460 | | 191 | | | | 65 | | | 10333 | | | 3970 | | | 162 | | | 105 | | | 5781 | | | 86 | | | 201 | | | 43 | | | 43 | | 42 | | 10055 | | 10055 | |
| **SD** | | | 13 | | | 2 | |  | | 265503 | | | 23 | | | 1328 | | | 1187 | | | 322 | | | 7 | | | 123 | | | 184 | | | 159 | | | 3789 | | 12 | | | | 39 | | | 628 | | | 225 | | | 7 | | | 6 | | | 449 | | | 9 | | | 18 | | | 6 | | | 6 | | 680 | | 680 | | 680 | |
| **%CV** | | | 12 | | | 24 | |  | | 7 | | | 40 | | | 25 | | | 28 | | | 16 | | | 8 | | | 6 | | | 7 | | | 6 | | | 7 | | 6 | | | | 60 | | | 6 | | | 6 | | | 5 | | | 6 | | | 8 | | | 11 | | | 9 | | | 14 | | | 14 | | 7 | | 7 | | 7 | |
| **Recovery relative to published values (%)** | | |  | | |  | |  | | 93^A^ | | | 96^B^ | | |  | | |  | | | 79^A^ | | | 82^B^ | | | 95^C^ | | | 79^A^ | | |  | | | 82^D^ | | 91^A^ | | | |  | | | 88^A^ | | | 83^A^ | | | 89^D^ | | | 37^E^ | | | 114^C^ | | |  | | |  | | |  | | |  | | 84^A^ | |  | | 92^A^ | |
| **Moth (PH_armig) in-house Std** | | | | | | | | | | | | | | | | | | | | | | | | | | | | | | | | | | | | | | | | | | | | | | | | | | | | | | | | | | | | | | | | | | | | | | | | | | | | | |
|  | **^7^Li** | | | | **^9^Be** | | **^27^Al** | | | | **^43^Ca** | | | | **^45^Sc** | | **^47^Ti** | | | | **^49^Ti** | | **^53^Cr** | | **^59^Co** | | **^60^Ni** | | | **^63^Cu** | | | **^65^Cu** | | | **^67^Zn** | | | | **^75^As** | | **^77^Se** | | | **^85^Rb** | | | **^88^Sr** | | | **^111^Cd** | | | **^115^In** | | | **^133^Cs** | | | **^137^Ba** | | | **^139^La** | | | **^140^Ce** | | | **^182^W** | | **^183^W** | | **^205^Tl** | | **^206^Pb** | | **^208^Pb** |
| **Mean** | 46 | | | | 2 | | 53975 | | | | 916110 | | | | 38 | | 14570 | | | | 5005 | | 397 | | 90 | | 247 | | | 19960 | | | 19864 | | | 368349 | | | | 206 | | 502 | | | 3746 | | | 11530 | | | 152 | | |  | | | 23 | | | 8544 | | | 18 | | | 44 | | | 99 | | 95 | | 1 | | 59 | | 54 |
| **SD** | 9 | | | | 1 | | 17348 | | | | 76637 | | | | 19 | | 3691 | | | | 713 | | 97 | | 14 | | 54 | | | 1710 | | | 1684 | | | 28300 | | | | 19 | | 71 | | | 204 | | | 945 | | | 19 | | |  | | | 4 | | | 689 | | | 5 | | | 11 | | | 11 | | 9 | | 1 | | 12 | | 22 |
| **%CV** | 20 | | | | 62 | | 32 | | | | 8 | | | | 50 | | 25 | | | | 14 | | 24 | | 16 | | 22 | | | 9 | | | 8 | | | 8 | | | | 9 | | 14 | | | 5 | | | 8 | | | 12 | | |  | | | 16 | | | 8 | | | 25 | | | 25 | | | 11 | | 10 | | 130 | | 20 | | 40 |

Asfaw, A., & Wibetoe, G. (2006). Dual mode sample introduction for multi-element determination by ICP-MS: the optimization and use of a method based on simultaneous introduction of vapor formed by NaBH4 reaction and aerosol from the nebulizer. *Journal of Analytical Atomic Spectrometry, 21*(10), 1027-1035.

Freitas, M., Pacheco, A., Bacchi, M., Dionísio, I., Landsberger, S., Braisted, J., & Fernandes, E. (2008). Compton suppression instrumental neutron activation analysis performance in determining trace- and minor-element contents in foodstuff. *Journal of Radioanalytical and Nuclear Chemistry, 276*(1), 149-156.

Reed, W. P. (1993). *Certificate of analysis - Standard Reference Material 1575, Pine needles*. Retrieved 6 June, 2011, from <http://ts.nist.gov/MeasurementServices/ReferenceMaterials/>

Saitoh, K., Sera, K., Gotoh, T., & Nakamura, M. (2002). Comparison of elemental quantity by PIXE and ICP-MS and/or ICP-AES for NIST standards. *Nuclear Instruments and Methods in Physics Research Section B: Beam Interactions with Materials and Atoms, 189*(1-4), 86-93.

Taylor, V., Evans, R., & Cornett, R. (2007). Rapid dissolution of large environmental samples for the determination of fission products. *Journal of Radioanalytical and Nuclear Chemistry, 274*(3), 545-553.
