# Supplementary material for: Isotopes and Trace Elements as Natal Origin Markers of Helicoverpa armigera – An Experimental Model for Biosecurity Pests
Source: PLoS One. 2014 Mar 24;9(3):e92384. doi: 10.1371/journal.pone.0092384 (PMC3963883; doi:10.1371/journal.pone.0092384)
Supplement: Table S3 — Typical instrument operating conditions of the Victoria University Nu MC-ICP-MS and the DSN-100 parameters used for Pb isotope analysis. (DOCX) [file pone.0092384.s004.docx]

**Table S3.** **Typical instrument operating conditions of the Victoria University Nu MC-ICP-MS and the DSN-100 parameters used for Pb isotope analysis**.

| **Multi-collector ICP-MS System** | Nu Instruments MC-ICP-MS |
| --- | --- |
| **High voltage** | 4000 V |
| **RF power** | ∼1300 W |
| **Coolant Ar flow** | ∼13 L/min |
| **Auxiliary Ar flow** | 0.9 L/min |
| **Variable slit width** | 0.03 mm |
| ***DSN-100 parameters*** | |
| **Membrane temperature** | 110 ºC |
| **Membrane gas flow** | 3.0-3.5 L/min |
| **Hot gas flow** | 23 PSI |
| **Spray chamber temperature** | 110ºC |
| **Sample uptake rate** | 100 µL/min |
| **Nebuliser pressure** | 31 PSI |
| **Sensitivity for Pb** | ∼300 V / ppm |
