# Supplementary material for: Isotopes and Trace Elements as Natal Origin Markers of Helicoverpa armigera – An Experimental Model for Biosecurity Pests
Source: PLoS One. 2014 Mar 24;9(3):e92384. doi: 10.1371/journal.pone.0092384 (PMC3963883; doi:10.1371/journal.pone.0092384)
Supplement: Table S4 — Pair-wise tests of regional differences for H. armigera populations, showing significant differences between the collection regions. Generated by PERMANOVA analyses of the multivariate datasets (using Euclidean distance resemblance matrices). † = p<0.10; * = p<0.05; ** = p<0.01. (DOCX) [file pone.0092384.s005.docx]

**Table S4.** **Pair-wise tests of regional differences for *H. armigera* populations, showing significant differences between the collection regions**.

|  | **Regions compared** | **t** | **P(perm)** | **Significance** |
| --- | --- | --- | --- | --- |
| **2008** | MC, BP | 1.2667 | 0.1329 | ns |
|  | **MC, AK** | **1.5664** | **0.0869** | **†** |
|  | **MC, NSW** | **1.9746** | **0.0278** | ***** |
|  | **MC, QLD** | **2.2951** | **0.0271** | ***** |
|  | BP, AK | 1.0209 | 0.2681 | ns |
|  | BP, NSW | 1.3102 | 0.1354 | ns |
|  | BP, QLD | 1.4058 | 0.1317 | ns |
|  | **AK, NSW** | **1.5538** | **0.0271** | ***** |
|  | **AK, QLD** | **1.5137** | **0.0278** | ***** |
|  | NSW, QLD | 1.2158 | 0.1445 | ns |
| **2009** | MC, BP | 1.2996 | 0.1372 | ns |
|  | **MC, AK** | **1.8044** | **0.0269** | ***** |
|  | **MC, NSW** | **1.5723** | **0.0299** | ***** |
|  | **MC, QLD** | **1.7403** | **0.0215** | ***** |
|  | BP, AK | 1.2023 | 0.1894 | ns |
|  | **BP, NSW** | **1.7564** | **0.0101** | ***** |
|  | **BP, QLD** | **1.6599** | **0.0182** | ***** |
|  | **AK, NSW** | **1.8677** | **0.0023** | ****** |
|  | **AK, QLD** | **2.0125** | **0.004** | ****** |
|  | **NSW, QLD** | **1.8334** | **0.0029** | ****** |

Generated by PERMANOVA analyses of the multivariate datasets (using Euclidean distance resemblance matrices). † = p <0.10; * = p <0.05; ** = p <0.01.
