# Supplementary material for: Isotopes and Trace Elements as Natal Origin Markers of Helicoverpa armigera – An Experimental Model for Biosecurity Pests
Source: PLoS One. 2014 Mar 24;9(3):e92384. doi: 10.1371/journal.pone.0092384 (PMC3963883; doi:10.1371/journal.pone.0092384)
Supplement: Table S5 — Relative contribution of the individual markers to the CAP regional grouping. Expressed as Pearson's correlation coefficient (i.e., linear measure of assocaition) between the individual markers (ignoring all others) and the CAP ordination axes within the mulitvariate data cloud. 2-sided significance test expressed as † = 10%; * = 5%; ** = 1%.; 2008 data df = 16, 2009 df = 18. 2009 data is an optimized suite. (DOCX) [file pone.0092384.s006.docx]

**Table S5.** **Relative contribution of the individual markers to the CAP regional grouping**.

2008

| **CAP axis** | **Li** | **Al** | **Sc** | **Cr** | **Mn** | **Ni** | **Zn** | **Ga** | **As** | **Rb** | **Sr** | **Cd** | **Cs** | **Ba** | **W** | **Pb** | **Rb/**  **Ba** | **Rb/Sr** | **Ba/Sr** | **Pb/Sr** | **d^2^H** | **^208^Pb/**  **^206^Pb** | **^207^Pb/**  **^206^Pb** |
| --- | --- | --- | --- | --- | --- | --- | --- | --- | --- | --- | --- | --- | --- | --- | --- | --- | --- | --- | --- | --- | --- | --- | --- |
| **CAP1** | -0.52* | -0.14 | 0.13 | 0.18 | -0.15 | -0.41† | 0.09 | 0.24 | -0.32 | 0.46† | -0.46† | 0.11 | 0.40† | 0.24 | 0.41† | 0.17 | 0.28 | 0.49* | 0.41 | 0.48* | -0.30 | -0.65 ** | 0.74 ** |
| **CAP2** | 0.13 | 0.42† | 0.25 | 0.54* | 0.30 | 0.04 | -0.06 | 0.58 ** | 0.26 | 0.49* | 0.45† | -0.39 | -0.17 | 0.58 ** | 0.54* | -0.16 | -0.32 | -0.15 | 0.02 | -0.56 ** | 0.67 ** | -0.02 | -0.08 |
| **CAP3** | -0.02 | -0.31 | -0.53* | -0.31 | -0.21 | -0.09 | -0.64 ** | -0.10 | -0.30 | -0.08 | 0.11 | 0.23 | -0.02 | -0.09 | 0.40† | 0.71 ** | -0.26 | -0.49* | -0.42 | 0.38 | -0.48* | -0.03 | -0.04 |
| **CAP4** | 0.05 | 0.07 | 0.18 | -0.16 | 0.15 | 0.22 | 0.03 | -0.09 | 0.03 | 0.28 | -0.18 | -0.15 | 0.61 ** | -0.08 | 0.18 | 0.25 | 0.30 | 0.49* | 0.03 | 0.29 | 0.02 | 0.52* | -0.20 |

2009

| **CAP axis** | **Ti** | **Co** | **Ni** | **Cu** | **As** | **Rb** | **Sr** | **Cd** | **Cs** | **Ba** | **La** | **Ce** | **Pb** | **Rb/Ba** | **Rb/Sr** | **Ba/Sr** | **Pb/Sr** | **d^2^H** | **^208^Pb/**  **^206^Pb** | **^207^Pb/**  **^206^Pb** | **^87^Sr/**  **^86^Sr** |
| --- | --- | --- | --- | --- | --- | --- | --- | --- | --- | --- | --- | --- | --- | --- | --- | --- | --- | --- | --- | --- | --- |
| **CAP1** | -0.31 | -0.28 | -0.10 | 0.30 | -0.09 | 0.27 | 0.09 | -0.05 | 0.10 | 0.08 | 0.17 | 0.15 | 0.44† | 0.29 | 0.26 | 0.16 | 0.32 | 0.69** | -0.28 | 0.19 | -0.85 ** |
| **CAP2** | 0.13 | 0.26 | -0.09 | -0.34 | -0.41† | -0.37 | 0.63** | -0.35 | -0.51* | 0.04 | 0.01 | -0.04 | -0.04 | -0.54* | -0.41† | -0.43† | -0.47* | 0.30 | 0.14 | -0.08 | -0.10 |
| **CAP3** | 0.40† | 0.53* | 0.55* | 0.20 | 0.28 | 0.37 | 0.05 | 0.24 | 0.02 | 0.73** | 0.71** | 0.73** | 0.19 | -0.07 | 0.31 | 0.48* | -0.09 | -0.02 | 0.42* | 0.31 | 0.09 |
| **CAP4** | -0.12 | -0.02 | 0.42† | 0.30 | 0.64** | -0.46* | 0.38† | 0.41† | -0.35 | 0.02 | 0.40† | 0.41† | 0.51* | -0.36 | -0.55* | -0.53* | -0.04 | 0.05 | -0.14 | 0.40† | -0.16 |

Expressed as Pearson’s correlation coefficient (i.e., linear measure of assocaition) between the individual markers (ignoring all others) and the CAP ordination axes within the mulitvariate data cloud. 2-sided significance test expressed as † = 10%; * = 5%; ** = 1%.; 2008 data df = 16, 2009 df = 18. 2009 data is an optimized suite.
