# Supplementary material for: Isotopes and Trace Elements as Natal Origin Markers of Helicoverpa armigera – An Experimental Model for Biosecurity Pests
Source: PLoS One. 2014 Mar 24;9(3):e92384. doi: 10.1371/journal.pone.0092384 (PMC3963883; doi:10.1371/journal.pone.0092384)
Supplement: Table S6 — H. armigera wing δ2H summary table. Showing regional δ2HM averages ± 1SD; values within a row that are followed by a different letter are significantly different (Fishers unrestricted LSD = 5%). (DOCX) [file pone.0092384.s007.docx]

**Table S6.** ***H. armigera* wing δ^2^H summary table**.

|  | MC | BP | AK | NSW | QLD |
| --- | --- | --- | --- | --- | --- |
| 2008 | -102.0±6.5 *^a^* | -84.2±8.0 *^bc^* | -79.3±7.3 *^b^* | -85.0±12.0 *^c^* | -72.8±6.1 *^d^* |
|  | (n=17) | (n=8) | (n=24) | (n=22) | (n=26) |
| Range | 25.7 | 25.9 | 24.8 | 42.0 | 26.5 |
| 2009 | -84.2±7.0 *^a^* | -61.5±10.6 *^b^* | -72.9±7.4 *^c^* | -73.6±8.4 *^c^* | -58.0±9.5 *^b^* |
|  | (n=43) | (n=26) | (n=59) | (n=54) | (n=33) |
| Range | 25.7 | 45.2 | 43.2 | 36.1 | 44.6 |

Showing regional δ^2^H_M_ averages ± 1SD; values within a row that are followed by a different letter are significantly different (Fishers unrestricted LSD = 5%).
