# Supplementary material for: Isotopes and Trace Elements as Natal Origin Markers of Helicoverpa armigera – An Experimental Model for Biosecurity Pests
Source: PLoS One. 2014 Mar 24;9(3):e92384. doi: 10.1371/journal.pone.0092384 (PMC3963883; doi:10.1371/journal.pone.0092384)
Supplement: Table S7 — Retrospective power analyses for H. armigera δ2H. To detect significant differences between the regional means (Δ‰), at a two-sided significance level of 0.05 with a power of 0.90 using a two-sample t-test, the calculated sample size (n) would be required for each sample. A standard deviation pooled across all regions was used in each power analysis. (DOCX) [file pone.0092384.s008.docx]

**Table S7.** **Retrospective power analyses for *H. armigera* δ^2^H**.

|  |  | **MC** | | **BP** | | **AK** | | **NSW** | |
| --- | --- | --- | --- | --- | --- | --- | --- | --- | --- |
|  |  | Δ‰ | **n** | Δ‰ | **n** | Δ‰ | **n** | Δ‰ | n |
| **2008** | **BP** | 18 | **5** |  |  |  |  |  |  |
|  | **AK** | 23 | **4** | 5 | **50** |  |  |  |  |
|  | **NSW** | 17 | **9** | -1 | **2166** | -6 | **59** |  |  |
|  | **QLD** | 29 | **3** | 11 | **10** | 7 | **21** | 12 | 15 |
| **2009** | **BP** | 23 | **5** |  |  |  |  |  |  |
|  | **AK** | 11 | **11** | -11 | **16** |  |  |  |  |
|  | **NSW** | 11 | **12** | -12 | **15** | -1 | **1325** |  |  |
|  | **QLD** | 26 | **4** | 4 | **134** | 15 | **8** | 16 | 8 |

To detect significant differences between the regional means (Δ‰), at a two-sided significance level of 0.05 with a power of 0.90 using a two-sample t-test, the calculated sample size (*n*) would be required for each sample. A standard deviation pooled across all regions was used in each power analysis.
