# Supplementary material for: Isotopes and Trace Elements as Natal Origin Markers of Helicoverpa armigera – An Experimental Model for Biosecurity Pests
Source: PLoS One. 2014 Mar 24;9(3):e92384. doi: 10.1371/journal.pone.0092384 (PMC3963883; doi:10.1371/journal.pone.0092384)
Supplement: Table S8 — H. armigera 87Sr/86Sr summary table. Showing regional 87Sr/86Sr averages ± 1SD; values within a row that are followed by a different letter are significantly different (Fishers unrestricted LSD = 5%). n = 6 for each region. (DOCX) [file pone.0092384.s009.docx]

**Table S8**. ***H. armigera* ^87^Sr/^86^Sr summary table**.

| Region | MC | BP | AK | NSW | QLD |
| --- | --- | --- | --- | --- | --- |
| Average | 0.709747 ± 0.00227 *^a^* | 0.709513 ± 0.00074 *^a^* | 0.708701 ± 0.00161 *^a^* | 0.712776 ± 0.00084 *^b^* | 0.706727 ± 0.00111 *^c^* |
| Median | 0.709420 | 0.709276 | 0.709180 | 0.712750 | 0.706543 |
| Range | 0.0062 | 0.0019 | 0.0042 | 0.0020 | 0.0034 |

Showing regional ^87^Sr/^86^Sr averages ± 1SD; values within a row that are followed by a different letter are significantly different (Fishers unrestricted LSD = 5%). *n* = 6 for each region.
