# Supplementary material for: Isotopes and Trace Elements as Natal Origin Markers of Helicoverpa armigera – An Experimental Model for Biosecurity Pests
Source: PLoS One. 2014 Mar 24;9(3):e92384. doi: 10.1371/journal.pone.0092384 (PMC3963883; doi:10.1371/journal.pone.0092384)
Supplement: Table S9 — Retrospective power analysis for the H. armigera 87Sr/86Sr data. To detect significant differences between the regional means (Δ), at a two-sided significance level of 0.05 with a power of 0.90 using a two-sample t-test, replication of the calculated n for each sample is required. (DOCX) [file pone.0092384.s010.docx]

**Table S9.** **Retrospective power analysis for the *H. armigera* ^87^Sr/^86^Sr data**.

|  | **MC** | | **BP** | | **AK** | | **NSW** | |
| --- | --- | --- | --- | --- | --- | --- | --- | --- |
|  | Δ | **n** | Δ | **n** | Δ | **n** | Δ | **n** |
| **BP** | -0.00023 | **1096** |  |  |  |  |  |  |
| **AK** | -0.00105 | **76** | -0.00081 | **52** |  |  |  |  |
| **NSW** | 0.003029 | **9** | 0.003263 | **3** | 0.004075 | **4** |  |  |
| **QLD** | -0.00302 | **9** | -0.00279 | **4** | -0.00197 | **12** | -0.00605 | **3** |

To detect significant differences between the regional means (Δ), at a two-sided significance level of 0.05 with a power of 0.90 using a two-sample t-test, replication of the calculated *n* for each sample is required.
