# Supplementary material for: Isotopes and Trace Elements as Natal Origin Markers of Helicoverpa armigera – An Experimental Model for Biosecurity Pests
Source: PLoS One. 2014 Mar 24;9(3):e92384. doi: 10.1371/journal.pone.0092384 (PMC3963883; doi:10.1371/journal.pone.0092384)
Supplement: Table S10 — Retrospective power analysis for the H. armigera 207Pb/206Pb data. To detect significant differences between the regional means (Δ), at a two-sided significance level of 0.05 with a power of 0.90 using a two-sample t-test, replication of the calculated n for each sample is required. (DOCX) [file pone.0092384.s011.docx]

**Table S10**. **Retrospective power analysis for the *H. armigera* ^207^Pb/^206^Pb data**.

|  |  | **MC** | | **BP** | | **AK** | | **NSW** | |
| --- | --- | --- | --- | --- | --- | --- | --- | --- | --- |
|  |  | Δ | **n** | Δ | **n** | Δ | **n** | Δ | **n** |
| **2008** | **BP** | 0.0006 | **6315** |  |  |  |  |  |  |
|  | **AK** | -0.00349 | **90** | -0.00409 | **246** |  |  |  |  |
|  | **NSW** | 0.0794 | **5** | -0.0788 | **6** | 0.0829 | **5** |  |  |
|  | **QLD** | 0.0024 | **352** | 0.0018 | **1767** | 0.0059 | **110** | -0.077 | **6** |
| **2009** | **BP** | 0.0073 | **208** |  |  |  |  |  |  |
|  | **AK** | 0.0128 | **59** | 0.0055 | **268** |  |  |  |  |
|  | **NSW** | 0.0033 | **3487** | -0.004 | **2177** | -0.0095 | **357** |  |  |
|  | **QLD** | 0.0067 | **169** | -0.0006 | **17071** | -0.0061 | **137** | 0.0034 | **2450** |

To detect significant differences between the regional means (Δ), at a two-sided significance level of 0.05 with a power of 0.90 using a two-sample t-test, replication of the calculated *n* for each sample is required.
